# Dual-transcriptomics on microdissected cells reveals functional specialisation of symbiont-bearing-cells and contrasted responses to nutritional stress in the cereal weevil

**DOI:** 10.1186/s40168-025-02164-0

**Published:** 2025-08-06

**Authors:** Nikoletta Galambos, Nicolas Parisot, Agnès Vallier, Claudia Bevilacqua, Séverine Balmand, Carole Vincent-Monégat, Rita Rebollo, Benjamin Gillet, Sandrine Hughes, Abdelaziz Heddi, Anna Zaidman-Rémy

**Affiliations:** 1 INSA Lyon, INRAE, BF2I, UMR203, 69621 Villeurbanne, France; 2 Université Paris-Saclay, INRAE, AgroParisTech, GABI, 78350 Jouy-en-Josas, France; 3 Institut de Génomique Fonctionnelle de Lyon (IGFL), CNRS UMR 5242, Ecole Normale Supérieure de Lyon, Université de Lyon, Lyon, France; 4 Institut Universitaire de France (IUF), Paris, France

**Keywords:** *Sitophilus oryzae*, Nutritional endosymbiosis, Mutualism, Bacteria-host interactions, Symbiosis, Insect, Laser-capture microdissection, Dual RNA-sequencing

## Abstract

**Background:**

Insects thriving on a nutritionally imbalanced diet often establish long-term relationships with intracellular symbiotic bacteria (endosymbionts), which complement their nutritional needs and improve their physiological performances. Endosymbionts are in host specialised cells, called the bacteriocytes, which in many insects group together to form a symbiotic organ, the bacteriome. The cereal weevil *Sitophilus oryzae* houses multiple bacteriomes at the adult mesenteric caeca.

**Results:**

Using microscopic cell imaging, we revealed that bacteriomes consist of several cell types, including progenitor cells, peripheral bacteriocytes, central bacteriocytes and epithelial cells. By combining laser capture microdissection and dual RNA-sequencing, we showed that both host cell types and their associated endosymbionts express distinct transcriptional profiles. The comparison between peripheral bacteriocytes and midgut cells from insects artificially deprived from endosymbionts (aposymbiotic) unravelled cellular pathways modulated by the presence of endosymbionts. The cell-specific response to endosymbionts in peripheral bacteriocytes includes a boost of fatty-acid and amino acid metabolisms. We found that central bacteriocytes overexpress transport and G-protein signalling-related genes when compared to peripheral bacteriocytes, indicating a signalling and/or transport function of these cells. Diet composition strongly impacts host and endosymbiont gene expression and reveals a molecular trade-off among metabolic pathways.

**Conclusions:**

This study provides evidence on how endosymbionts interfere and enhance metabolic performances of insect bacteriocytes and highlights key genes involved in the bacteriocyte differentiation and metabolic pathways.

Video Abstract

**Supplementary Information:**

The online version contains supplementary material available at 10.1186/s40168-025-02164-0.

## Introduction

Nutritional endosymbiosis is widespread in insects thriving on imbalanced diets and involves a tight functional integration of endosymbionts within the host metabolic pathways [21]. Endosymbionts are housed within specialised host cells, called the bacteriocytes, which group together in several insects to form a symbiotic organ, the bacteriome, where the nutrient exchange takes place [11, 63]. While their evolutionary origin remains unclear, bacteriomes evolved independently in diverse insect orders [57], thus showing a remarkable diversity in their number, location, shape and structural organisation [50]. Despite their central role in metabolic cooperation, little is known about how bacteriomes are organised and function in the context of the interactions between symbiotic partners.

The association between the cereal weevil *Sitophilus oryzae* and the obligate endosymbiont *Sodalis pierantonius* represents a remarkable model system with unique characteristics. Firstly, *S. pierantonius* is the only endosymbiont associated with *S. oryzae*, offering a simple binary system to study the interactions [14, 34, 43, 65]. Secondly, aposymbiotic weevils (artificially deprived of endosymbionts) can be obtained under laboratory conditions, allowing for comparative studies with symbiotic individuals [62]. Lastly, cereal weevils established endosymbiosis with *S. pierantonius* relatively recently, around 30,000 years ago [14, 34, 43, 65]. Unlike ancient endosymbioses where bacterial genomes lost genes that are not required for the intracellular lifestyle [15, 57], *S. pierantonius* genome encodes type III secretion system (T3SS), flagellum and cell wall components (lipopolysaccharides, phospholipids) related genes [65]. As for *S. oryzae*, the immune Toll and IMD pathways are conserved [66] and *S. pierantonius* can be recognised by the host immune system when outside the bacteriome [4]. In contrast, in the pea aphid (*Acyrthosiphon pisum*) and *Buchnera aphidicola* endosymbiosis, established around 100 million years ago, the host lacks IMD pathway [75] and endosymbiont genome is highly reduced, retaining genes indispensable for biosynthesis of amino acids for the host, but lacking genes for defence and for the biosynthesis of some of the cell surface components [69].

*S. oryzae* feeds entirely on cereal grains, a diet rich in carbohydrates but lacking many metabolic components, including vitamins and amino acids. Endosymbionts are thought to synthesise these metabolites and supplement them to the host [30, 65, 66, 79–81]. As a result, the provision of these metabolites positively impacts host fitness traits, including reproduction, developmental time and flight ability [31, 35, 79]. Endosymbionts also interfere with cuticle synthesis following insect metamorphosis by providing the host with phenylalanine and tyrosine, necessary for cuticular sclerotization and tanning [79].

The larval stages of *S. oryzae* harbour a single bacteriome organ located at the fore-midgut junction [52]. During metamorphosis, this single bacteriome dissociates and releases many bacteriocytes that migrate along the midgut. At several locations of the adult midgut mesenteric caeca, multiple bacteriomes are differentiated, following endosymbiont infection of insect progenitor cells [52]. In aposymbiotic insects, mesenteric caeca are formed but without any bacteriome. The symbiotic rearrangement and increase in bacteriome number during metamorphosis help sustaining increased metabolic requirements at initial adult stages, including the cuticle synthesis [52, 79]. In parallel to bacteriome multiplication, endosymbiont load drastically increases in young adults, then bacteria are eliminated and recycled following the insect cuticle achievement, while they are maintained in ovaries from where they are transmitted to the next generation [27, 79].

Here, by using different histological approaches, we describe the different cell types that constitute the adult bacteriome, along with the midgut mesenteric caeca of aposymbiotic weevils. We have applied a dual RNA-sequencing approach on microdissected cells to analyse the gene expression and regulation of both host and endosymbiont in these cell types.

We show that the adult bacteriome and aposymbiotic mesenteric caecum are formed with morphologically distinct cell types in which both the host and the endosymbiont express different transcriptional programs. Our results provide evidence that these distinct transcriptional programs transform midgut cells into interkingdom “metabolic factories” and elucidate how metabolic capacities are improved in symbiotic insects, when compared to aposymbiotic ones.

To better understand how host and endosymbiont cope with changes in diet composition, we applied nutritional stress, including starch-exclusive diet and starvation, and analysed the transcriptional response of both symbiotic partners. We demonstrate that both host and endosymbiont strongly respond to nutritional stress. In the starch-exclusive diet, endosymbionts induce genes involved in amino acid metabolism. Under starvation, the pathways encoding the metabolism of amino acids and fatty acids are negatively affected in both host and endosymbionts.

## Material and methods

### Insect rearing and staging

Insects were reared on wheat grains at 27.5 °C and at 70% relative humidity. The Bouriz strain of *S. oryzae* was used, as it is free of any facultative symbionts and harbours *S. pierantonius,* exclusively. The full procedure to obtain aposymbiotic insects through heat-treatment is described elsewhere [52, 62]. To obtain 3-day-old adult insects, pupae were collected and transferred to well plates until adults emerged as described by Dell’Aglio et al. [18]. Adults able to walk were collected daily and were allowed to feed on a grain diet, starch diet (Stijfsel Remy, Belgium) or kept under starvation (insects were kept under same rearing condition but deprived from any food source) for 2 days before experiments [18]. Starch diet and starvation, representing pure carbohydrate diet and nutrient scarcity, respectively, were chosen to study the plasticity of the system.

### Preparation of frozen midguts for light microscopy

Midguts of 3-day-old symbiotic and aposymbiotic adults were dissected in diethylpyrocarbonate-treated buffer A (25 mM KCl, 10 mM MgCl_2_, 250 mM sucrose, 35 mM Tris/HCl, pH = 7.5). Fifteen midguts of both symbiotic and aposymbiotic insects were frozen in tissue freezing medium (General data company, OH, USA) and stored at − 80 °C. Cryosectioning was performed at − 20 °C in an RNA-free, RNAzap and 70% ethanol-treated Shandon cryostat (Thermo Scientific, Waltham, MA, USA). Eight-µm-thick midgut sections both from symbiotic and aposymbiotic insects were collected on poly-lysine glass slides (Thermo Scientific) and were stored at − 80 °C until further processing.

### Cresyl violet staining

Midguts sections on poly-lysine glass slides were dehydrated in 50% ethanol for 20 s, coloured in 1% cresyl violet in 50% ethanol for 25 s, washed in 50% ethanol for 20 s, 75% ethanol for 30 s, twice in 95% ethanol for 30 s and twice in 100% ethanol for 30 s. All ethanol residues were removed by washing the slides twice in Diasolv for one min and mounted in Diamount mounting medium (Diapath, Martinengo, Italy). Images were taken using an Olympus IX 81 microscope with the XC50 colour camera.

### FISH staining

Midgut sections on poly-lysine glass slides were fixed in 4% paraformaldehyde for 10 min and were rinsed two times in 1 X PBS. Slides were dehydrated in increasing concentrations of ethanol solution (70%, 95% and two times 100%, two min each step). Deproteinisation of slides was performed in hydrochloric acid 0.01 N with pepsin 0.1 mg/ml for 10 min at 37 °C followed by a quick rinse in PBS and dehydration in increasing concentrations of ethanol solution (70%, 95% and two times 100%, two min each step). Prehybridisation was carried out for 30 min at 45 °C in a prehybridisation buffer [79% of hybridisation buffer (NaCl 0.9 M, Tris 20 mM, EDTA 5 mM, pH 7.2), 20% of Denhardt (10% w/v Ficoll, 10% w/v polyvinylpyrrolidone, 10% w/v bovine serum albumin), and 1% SDS], prior to hybridisation at 45 °C in a hybridisation buffer with 10 µM TAMRA-labelled probe targeting the 16S rRNA gene of *S. pierantonius* (5′-/56-TAMN/ACC-CCC-CTC-TAC-GAG-AC-3′ Integrated DNA Technologies, IA, USA). After 3 h of incubation, sections were washed with hybridisation buffer with SDS 0.1%, rinsed in PBS and distilled water. Slides were allowed to dry before being mounted in DAPI-added PermaFluor™ Aqueous Mounting Medium (Thermo Scientific). Slides were then kept at 4 °C in the dark until observation under a fluorescence microscope (Leica Thunder, Wetzlar, Germany), using specific emission filters: ET630/75 for the red signal (probe staining), D470/40 for the blue signal (DAPI) and LP527/30 for the green signal (unspecific autofluorescence from tissues). Images were captured using a K3M monochrome camera and the LasX software (Leica) and representative images were selected. Images were cropped and light/contrast balance improved in post process.

### TEM: fixation using high-pressure freezing and observation

Midguts were dissected as described above. For electron microscopy (EM) observations, samples were first fixed using the High-pressure Freezing (HPF) technique: tissues were freshly dissected in buffer A solution (25 mM KCl, 10 mM MgCl_2_ 250 mM sucrose, 1% DEPC, 35 mM Tris–HCl pH 7.5), transferred to gold-plated copper specimen carriers (cylinder-shaped indentation on both sides, diam. 2 × 0.2 mm; Leica) and coated with 1-hexadecene. HPF was performed using the Leica HPM100. The freeze-substitution solution (FS) was composed of acetone with OsO4 1%, uranyl acetate 0.25%, glutaraldehyde 0.5% and water 1.5%. In the AFS2 FS apparatus (Leica), the samples in the carriers were left in the FS solution for about 30 h at − 90 °C, then the temperature was slowly increased to − 30 °C at a rate of 5 °C/h. They were left for another 24 h at − 30 °C until the temperature was again increased at room temperature at a rate of 10 °C/h. During this temperature rise, the FS solution was replaced by several acetone baths to rinse the samples which were also removed from their carrier. At room temperature, the acetone was gradually replaced by Epon, until the final embedding in Epon for 72 h at 60 °C. Semi-thin sections. (200–300 nm) were cut from the Epon block with a Leica UC7 ultramicrotome, deposited on glass slides and stained with toluidine blue. Additionally, ultrathin 70-nm-thick sections were cut and deposited on copper/formvar grids. Once sections were dried, they were contrasted with lead citrate before observation under a Philips CM120 transmission electron microscope.

### TEM: chemical fixation and observation

Midguts were dissected as described above and fixed with a solution of glutaraldehyde 3% in sodium cacodylate buffer (0.1 M) for 48 h. Fixative was replaced with several changes of sodium cacodylate buffer (0.2 M) and post-fixed in osmium tetroxide 1% for 1 h. After water washing, samples were dehydrated through a graded ethanol series, from ethanol 30% to absolute ethanol. Samples were then moved into a mix of absolute ethanol/propylene oxide (v/v) for 1 h. Substitution was performed in an epoxy resin (EPON) with propylene oxide (v/v). Afterwards, tissues were impregnated overnight with EPON and embedded in EPON with benzyl di-methyl amine (BDMA) 1.7% at 60 °C for one week. Resulting resin blocks were cut into 70-nm-thick sections using an ultramicrotome. Sections were placed on 100 mesh copper/formvar grids and dried. Sections were contrasted with uranyl acetate 7% in methanol for 7 min, then with lead citrate for 7 min. Sections were air dried and observed with a JEOL 1400 transmission electron microscope.

### Preparation of paraffin embedded samples and immunostaining

Midguts were dissected as described above and fixed in paraformaldehyde 4% in PBS. Samples were then rinsed and dehydrated through a graded ethanol/H2O series and moved to 1-butanol at 4 °C. Afterward tissues were impregnated and embedded in melted Paraplast Plus (Leica Biosystems Paraffin products). Tissue Sects. (3 µm thick) were obtained using a Thermo Scientific Microm HM340E microtome. Sections were placed on poly-lysine-coated slides, dried overnight in a 37 °C oven and stored at 4 °C before immunostaining. Paraffin sections were dewaxed in two baths of methylcyclohexane for 10 min, rinsed in ethanol 99% and rehydrated through an ethanol gradient to PBS. All slides were incubated with 1% BSA in PBS during 30 min prior primary antibody incubation (Table S1), overnight at 4 °C. Primary antibodies were diluted in PBS containing 0.1% BSA. PBS with BSA 0.1% without antibody was used as negative control. Afterward, sections were washed with PBS containing Tween 0.2%. Then samples were incubated with secondary antibodies, diluted in 0.1% BSA (Table S1), for 1 h at room temperature. Sections were then washed with PBS-Tween, rinsed with PBS and with several baths of water. Then sections were left to dry on the bench and mounted using Fluoro Gel with DABCO™ mounting medium in addition with 4,6-diamidino-2-phenylindole (DAPI) for nuclear staining (3 µg per ml of medium). Images were taken with an epifluorescence microscope (Olympus IX81), using appropriate filters for antibody staining, DAPI, and unspecific autofluorescence from tissues, or using a confocal microscope (Zeiss LSM800) using 405–488-561 lasers.

### Preparation of frozen midguts for laser capture microdissection

Midguts were dissected and cryosectioning was performed as described above. Eight-µm-thick midgut sections both from symbiotic and aposymbiotic insects were collected on Arcturus PEN membrane RNAse-free glass slides (Excilone, France) and were fixed immediately in 75% cold ethanol in the cryostat before further processing. Midguts were dehydrated and stained with cresyl violet as described above. All ethanol residues were removed by washing the slides twice in xylene for 1 min.

### Laser capture microdissection

The different cell types studied of adult bacteriomes (peripheral bacteriocytes, central bacteriocytes, epithelial cells) and aposymbiotic mesenteric caeca (caeca cells and epithelial cells) were identified under the optical microscope and laser capture microdissection (LCM) was carried out using the XT® Arcturus Technologies microdissection system (Excilone, France). Infrared laser (IR) and ultraviolet laser (UV) was used to capture and stick the different cell types in Arcturus CapSure® LCM Macro Caps (Excilone, France) and to remove non-target tissue, respectively. For downstream analysis, three to 12 caps per cell type were prepared and stored at − 80 °C.

### RNA extraction, library preparation and dual RNA-sequencing

Total RNA was extracted using the Picopure™ RNA Isolation kit (Thermo Scientific). Total RNA was eluted in an 11 µl elution buffer and RNA quality and quantity were assessed using an Agilent 2100 Bioanalyzer (Agilent Technologies, Palo Alto, CA) with an RNA6000 Pico Lab Chip. For each cell type, three replicates (pool of one to four caps) were analysed. RNA samples of 4–15.8 ng of the total RNA were subjected to dual RNA-seq library construction, using the Ovation® Solo™ Universal RNA-seq protocol (Tecan, Switzerland). Depletion of rRNA was performed with a total of 170 rRNA depletion probes designed for *S. oryzae* (5.8S, 18S, 28S and mitochondrial 12S and 16S) and for *S. pierantonius* rRNA sequences (5S, 16S and 23S) using the Ovation® Solo™ Universal RNA-seq protocol (Tecan, Switzerland), according to the manufacturer’s instructions. Paired-end reads of 75 nucleotides were obtained using an Illumina NextSeq 500 instrument (Illumina) at the sequencing platform of the Institut de Génomique Fonctionnelle de Lyon, École Normale Supérieure de Lyon, France and sequences were deposited at the Sequence Read Archive (SRA) of the National Center for Biotechnology Information (https://www.ncbi.nlm.nih.gov/sra) under the BioProject accession number PRJNA1195835.

### Bioinformatic analysis and identification of differentially expressed genes

Raw reads were cleaned and filtered using TRIMGALORE v0.6.6 (https://github.com/FelixKrueger/TrimGalore) with default parameters. Filtered read pairs were aligned and counted using STAR v2.7.10a [20] and BOWTIE v.2.4.5 [42] to *S. oryzae* genome (GCF_002938485.1) and to *S. pierantonius* genome (GCF_000517405.1), respectively. Shared reads between the two genomes were filtered using SAMTOOLS v1.15 [17] and PICARD v2.23.0 (https://broadinstitute.github.io/picard/). Gene counts of unambiguously mapped read pairs were obtained using HTSEQ-COUNT v1.99.2 with union mode [2]. Differentially expressed genes (DEGs) were identified using DESEQ2 v1.42.0 [47] to perform pairwise comparisons. *S. oryzae* genes were considered differentially expressed when the FDR adjusted *p*-values (P-adj) was lower than 0.05 and were selected for further analysis when log2 fold-change (Log2FC) was greater than 1 (for upregulated genes) or smaller than −1 (for downregulated genes). Since bacterial RNA was present in smaller quantities than the insect’s RNA, *S. pierantonius* genes were considered differentially expressed when the P-adj was lower than 0.1. Principal component analysis (PCA) was performed based on normalised counts using DESEQ2 and GGPLOT2 v3.5.0 [82]. Heat map diagrams of fold change values of DEGs were built using the Java Treeview 1.2.0 (http://jtreeview.sourceforge.net). Gene Ontology (GO) terms, Clusters of Orthologous genes (COG) terms and Kyoto Encyclopedia of Genes and Genomes (KEGG) pathway terms were annotated with EGGNOG-MAPPER v2.1.9 [12]. GO enrichment analysis were performed using TOPGO v2.54.0 [1] with the “classic” algorithm for *S. pierantonius* and g:profiler (v. e111_eg58_p18_30541362) with default parameters [41] for *S. oryzae*. Schematic representation of host cell types and *S. pierantonius* cells were generated with Biorender (https://biorender.com/).

## Results and discussion

### The adult bacteriome organ is formed of distinct cell types

To address the bacteriome morphological and functional organisation, we imaged three-day-old symbiotic and aposymbiotic adult insects. This corresponds to the developmental stage when endosymbiont number exponentially increases and insects’ cuticle synthesis is being completed [79], allowing to observe the organ’s functional organisation at a critical stage for metabolite production and nutrient exchanges.

**Fig. 1:**
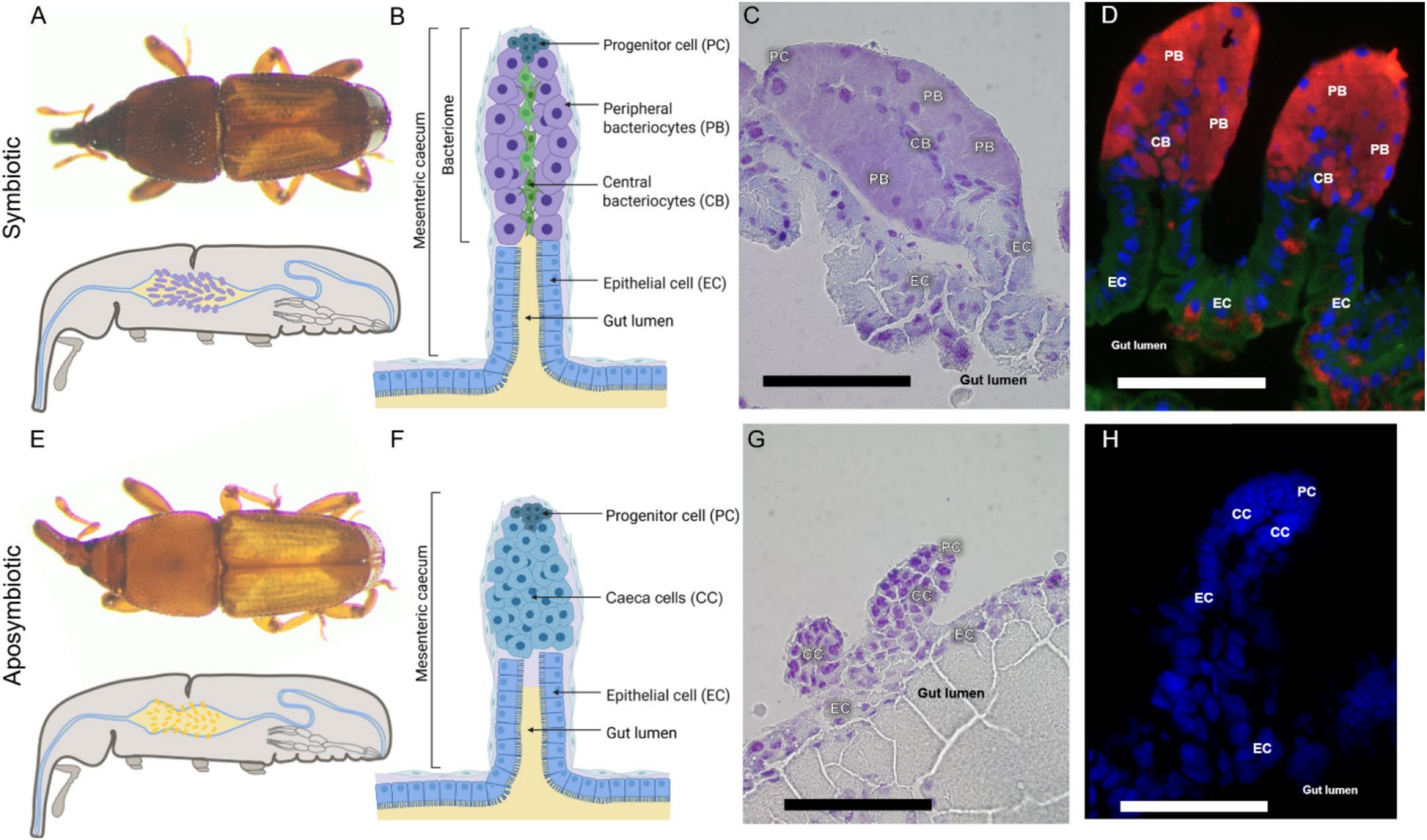
The adult bacteriome organ is formed of distinct cell types. **A** Symbiotic *Sitophilus oryzae* adult (top) and schematic representation of a sagittal section (bottom). **B** Schematic illustration of a midgut mesenteric caecum with a bacteriome at its apex from symbiotic *Sitophilus oryzae* adult. **C–D** Mesenteric caecum from symbiotic insects stained with cresyl violet (**C**) and FISH probe targeting the 16S rRNA of *Sodalis pierantonius* (**D**). In symbiotic adults four types of cells are observed: progenitor cells (PC), peripheral bacteriocytes (PB), central bacteriocytes (CB) and epithelial cells (EC) forming the basis of the mesenteric caecum. In symbiotic insects, endosymbionts can be seen in both types of bacteriocytes (light purple in **C**; red in **D**) and some epithelial cells also harbour endosymbionts (red in **D**). **E** Aposymbiotic *Sitophilus oryzae* adult (top) and schematic representation of a sagittal section (bottom). **F** Schematic illustration of a midgut mesenteric caecum from aposymbiotic *Sitophilus oryzae* adult. **G–H** Mesenteric caecum from aposymbiotic insects stained with cresyl violet (**G**) and FISH probe targeting the 16S rRNA of *Sodalis pierantonius* (**H**). In aposymbiotic adults three cell types are observed: progenitor cells (PC), caeca cells (CC) and epithelial cells (EC). Scale bars: 100 µm

Adult weevils harbour their endosymbionts in bacteriomes located at the apex of the numerous midgut mesenteric caeca (Fig. 1A; [79]). We used histological staining on tissue sections and Fluorescence in situ Hybridisation (FISH) using probes targeting the endosymbiont 16S rRNA to analyse in more detail the bacteriome cellular organisation. A clear morphological distinction among cells was observed, revealing a bacteriome organisation into several distinct cell types (Fig. 1B–D). Progenitor cells that are located at the apex of bacteriomes have previously been described as actively dividing [52]. Peripheral bacteriocytes are large cells (ranging between 20 and 40 µm) that fill the most of the bacteriome volume and house the great majority of the endosymbionts. They are located at the most outside part of the cylinder-shaped organ and have no interface with the gut lumen, hence lacking a direct access to the digested nutrients. In contrast, central bacteriocytes are smaller than peripheral bacteriocytes, although they also harbour endosymbionts. Based on FISH images (Fig. 1D), their central location inside the organ seems to provide the most basal of these central bacteriocytes with a direct interface with the gut lumen (at the basis of the caeca). Moreover, the central location of these cells inside the caeca also gives them direct contact with the peripheral bacteriocytes. The caeca bases are constituted of epithelial cells. To note, FISH images revealed that some of these epithelial cells harbour bacteria, although in lower amounts than the specialised bacteriocytes (Fig. 1D).

To understand the impact of endosymbionts on the insect cellular differentiation, we observed the cellular organisation in three-day-old aposymbiotic insects. As their symbiotic counterparts, aposymbiotic insects harbour progenitor cells at the apex, which have been described to proliferate (Fig. 1E; [52]). As expected, no bacteriocyte-looking cells are present (Fig. 1F–H). However, the caeca are not uniquely constituted of epithelial cells but present another type of cells. These round cells deprived of endosymbionts are morphologically different from both epithelial cells and bacteriocytes, and are herein referred to as “caeca cells” (Fig. 1F–H).

The observation of distinct morphological cell types in symbiotic insects reveals a functional cellular specialisation inside the bacteriome. Comparison with aposymbiotic insects indicated that the cell differentiation is impacted by the endosymbiont presence. However, the aposymbiotic caeca cell is morphologically distinct from both epithelial cells and bacteriocytes, suggesting that while endosymbiont presence likely induces host cell differentiation, it is not the only factor influencing their cell fate. This result challenges previous assumption that bacteriocytes are cells whose epithelial destiny has been modified by the presence of endosymbionts [52].

### The cell types constituting the bacteriome of symbiotic insects and the mesenteric caeca of aposymbiotic insects show distinct transcriptomic signatures

The observation of distinct morphological features in the cell types forming the bacteriome of symbiotic insects, as well as in the mesenteric caeca of aposymbiotic insects, raises three key questions: (1) whether these cells, characterised by various locations and morphologies, perform different functions within the organ; (2) whether the presence of endosymbionts influences gene expression in these cells; and (3) whether and how the endosymbionts express different genes depending on their location within a given cell type.

To explore these questions, we used a laser capture microdissection to specifically sample peripheral bacteriocytes, central bacteriocytes and epithelial cells from 3-day-old symbiotic insects, as well as caeca cells and epithelial cells from aposymbiotic insects. An example of LCM capture can be seen on Figure S1A. We then performed dual RNA-seq on these samples to determine the transcriptomic signatures of both host and endosymbionts in the different cell types.

Sequencing results are summarised in Table S2. Briefly, from 68.4 to 99.4 million weevil reads and 0.0 to 22.9 million endosymbiont reads were obtained for each replicate of the different cell types (Table S2). Endosymbiont reads account for 22.18 to 23.55% of total reads in peripheral bacteriocytes, correlating with the high number of endosymbionts in this cell type (Figure S1B and Table S2). In central bacteriocytes, endosymbiont reads ranged from 4.45 to 6.09%, attesting the presence of endosymbionts, though in lower numbers, when compared to peripheral bacteriocytes. In epithelial cells, endosymbiont reads were much lower (0.53 to 1.20%), in accordance with the limited bacterial presence observed via fluorescence microscopy (Fig. 1D; Figure S1B and Table S2). Expression levels of *S. oryzae* and *S. pierantonius* genes are summarised in Table S3 and S4, respectively.

In symbiotic insects, weevil normalised gene counts revealed a clear distinction between bacteriocytes and epithelial cells, in line with their morphological differences (Fig. 1B–D and Figure S1C). Both types of bacteriocytes (central and peripheral) cluster closely in the PCA, indicating that their expression profiles are more similar than with other cells. This confirms that central bacteriome cells are bacteriocytes, albeit less infected and morphologically distinct from the peripheral ones (Figure S1C). Interestingly, in aposymbiotic insects, expression profiles of caeca cells and epithelial cells are distinct. This indicates that even in the absence of endosymbiont infection, these cells differentiate into a different cell type than epithelial cells (Fig. 1G–H and Figure S1C). However, PCA also shows clear differences in the expression profiles of symbiotic bacteriocytes and aposymbiotic caeca cell types; the latter demonstrating higher similarities with epithelial cells than with bacteriocytes (Figure S1C). This suggests a strong impact of endosymbiont presence on the regulation of gene expression in midgut cells. In contrast, epithelial cells show a similar transcriptomic landscape whether sampled from symbiotic or aposymbiotic insects (Figure S1C).

Strikingly, endosymbiont expression profiles did not show any difference between peripheral and central bacteriocytes (Figure S1D), indicating a similar bacterial gene regulation in these two types of host cells. However, PCA revealed differences in the endosymbiont expression profiles whether located in the bacteriocytes or epithelial cells (Figure S1D), which indicates that endosymbionts undergo a distinct transcriptional program when infecting different host cells.

These data indicate that the different cell types forming the bacteriome likely perform different functions within the organ given their distinct transcriptomic profiles; that the presence of endosymbionts influences their gene expression, as demonstrated by comparing with endosymbiont-free cells; and that endosymbionts express different genes depending on their location within a given cell type.

### The presence of endosymbiotic bacteria triggers a metabolic upgrading of the midgut mesenteric caeca

To further analyse the effect of endosymbiont presence on host gene expression, we identified differentially expressed genes (DEGs) in the pairwise comparisons between peripheral bacteriocytes and aposymbiotic caeca cells, and between central bacteriocytes and aposymbiotic caeca cells.

**Fig. 2:**
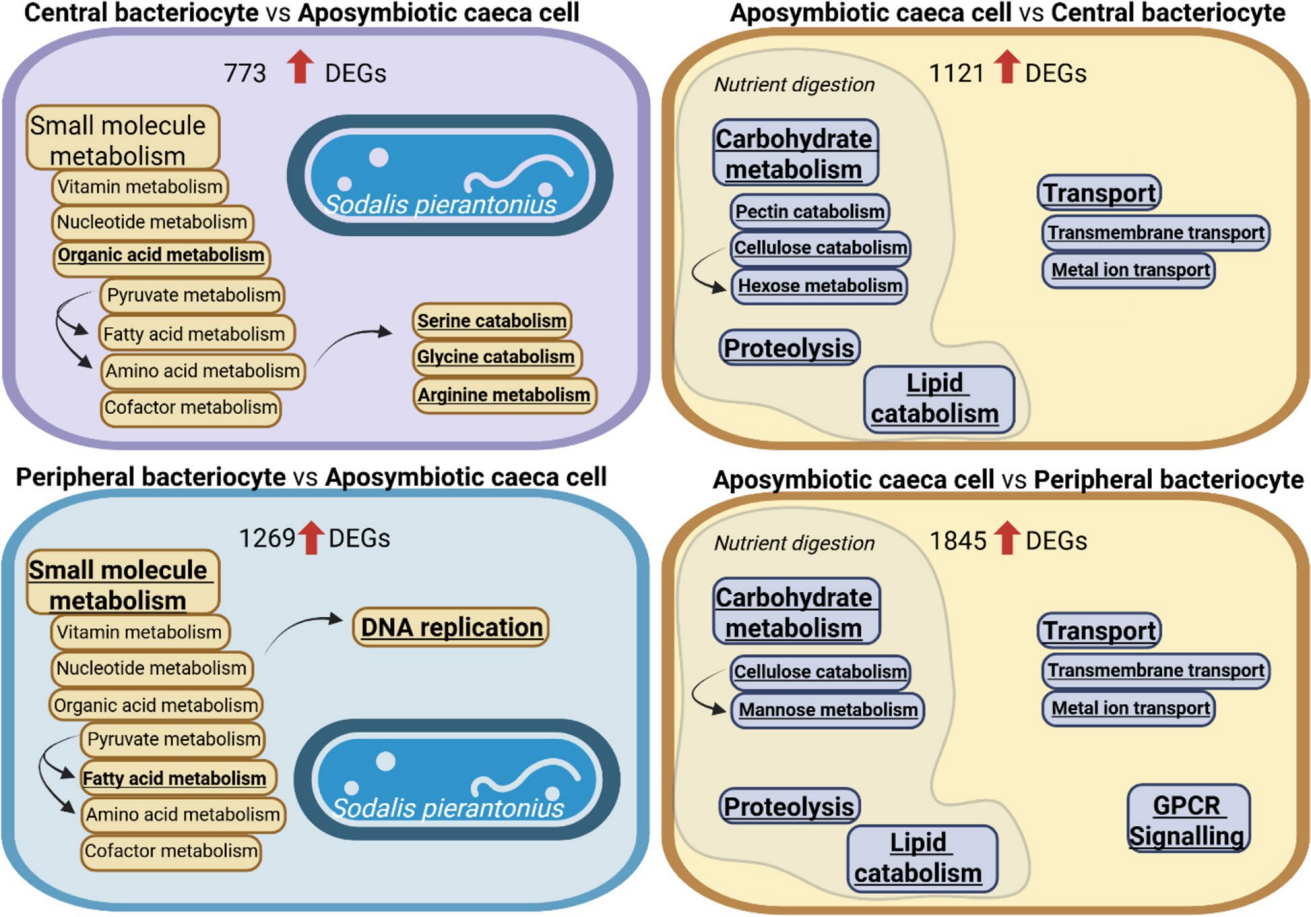
The endosymbiont *Sodalis pierantonius* affects important biological processes in *Sitophilus oryzae* bacteriocytes. Enriched GO terms in upregulated host genes in central and peripheral bacteriocytes compared to aposymbiotic caeca cells (left) and in aposymbiotic caeca cells compared to central and peripheral bacteriocytes (right). Underlined GO terms indicate significant functional enrichment (FDR adjusted-*P* ≤ 0.05)

In aposymbiotic caeca cells when compared to peripheral and central bacteriocytes, 1845 and 1121 weevil genes were upregulated, respectively (Figure S2A and Table S5). A significant enrichment of GO terms related to carbohydrate metabolism, including pectin and cellulose catabolism and hexose metabolism, proteolysis, lipid catabolism and transmembrane transport, was found for upregulated DEGs in aposymbiotic caeca cells as compared to bacteriocytes (Fig. 2 and Table S6). In particular, we found that genes encoding enzymes involved in the carbohydrate catabolism (*e.g*. α-amylase, α-glucosidase, α-L-fucosidase, β-galactosidase, α-mannosidase, β-mannosidase, endoglucanase, maltase, polygalacturonase, pectinesterase), proteolysis (*e.g.* carboxypeptidase, cathepsin, trypsin) and lipid degradation (*e.g.* acyl-coenzyme A oxidase, ceramidase, sphingomyelin phosphodiesterase) were upregulated in aposymbiotic caeca cells, when compared to bacteriocytes. The lower expression of these genes in bacteriocytes could indicate an endosymbiont contribution to nutrient digestion, whereas in the absence of this contribution, the host cells would need to express them more. Indeed, while the genome of *S. pierantonius* is known to have lost many genes, it retained maltodextrin phosphorylase (malP; SOPEG_0299), which allows starch degradation [65]. An alternative explanation could be that in the absence of endosymbionts, and therefore with lower metabolic efficiency, the insect must catabolise more carbohydrates and lipids to fulfil its metabolic requirement. Likewise, Baker [5] showed evidence that endosymbionts might assist host cholesterol utilisation [5].

In peripheral and central bacteriocytes, 1269 and 773 weevil genes were upregulated as compared to aposymbiotic caeca cells, respectively (Figure S2A and Table S5). A significant enrichment of GO terms related to small molecule metabolism, including fatty acid derivative metabolic process and DNA replication was found for upregulated DEGs in peripheral bacteriocytes when compared to aposymbiotic caeca cells (Fig. 2 and Table S6). A significant enrichment of GO terms related to organic acid metabolic process, including arginine metabolic process, glycine and serine catabolic processes was found in upregulated DEGs in central bacteriocytes, when compared to aposymbiotic caeca cells (Fig. 2 and Table S6).

**Fig. 3:**
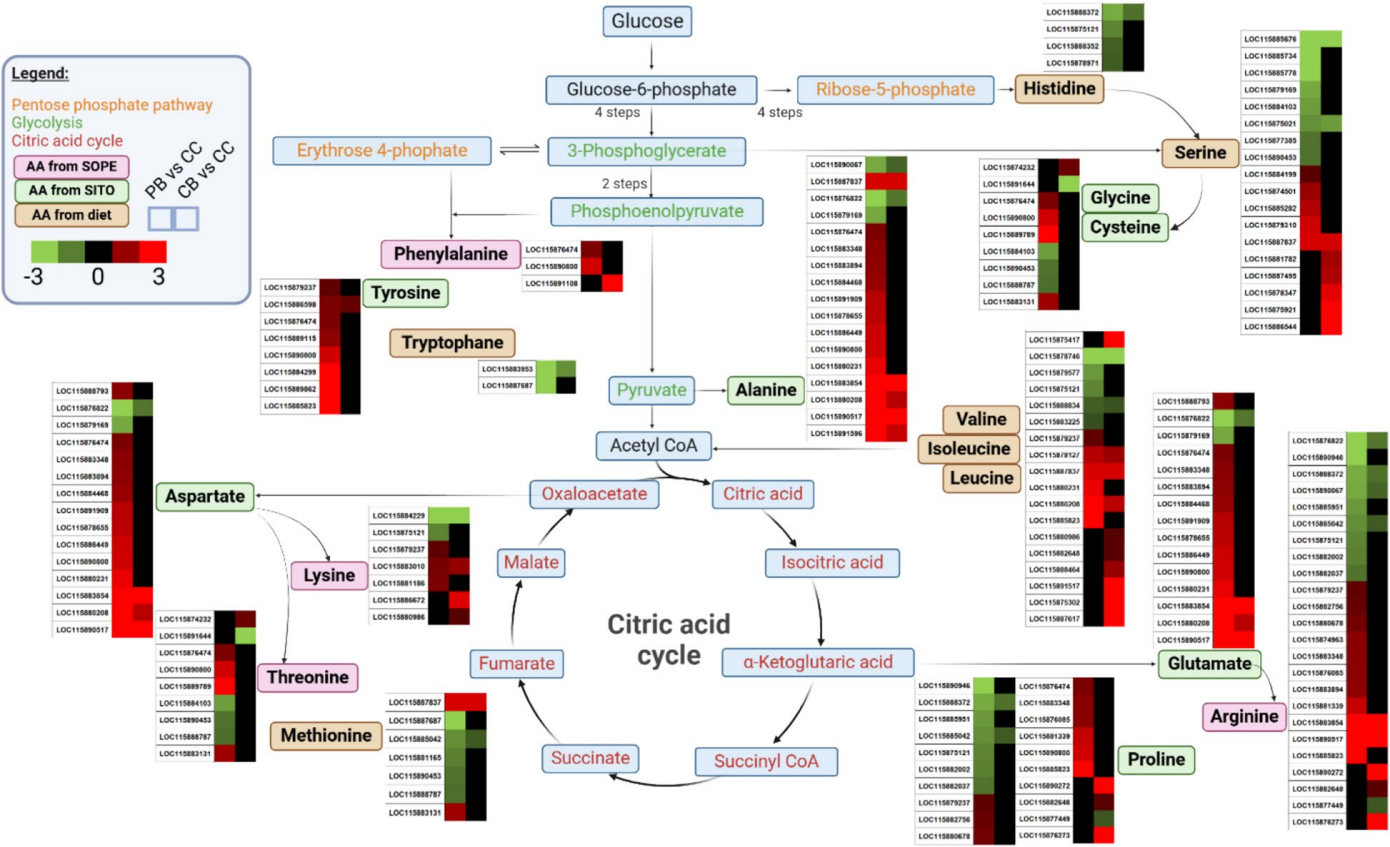
Amino acid metabolism is affected by the presence of the endosymbiont. Differentially expressed genes from *Sitophilus oryzae* related to amino acid (AA) metabolism, identified in the pairwise comparisons between peripheral (PB) and central bacteriocytes (CB), compared with aposymbiotic caeca cells (CC). For each gene, two squares represent Log_2_-transformed fold change values of peripheral (left) and central bacteriocytes (right) compared to aposymbiotic caeca cells, according to the colour scale reported. Pink, green and brown boxes indicate amino acids provided by *Sodalis pierantonius* (SOPE)*, Sitophilus oryzae* (SITO) and obtained from diet, respectively

Interestingly, in both peripheral and central bacteriocytes, we found amino acid metabolism-related genes upregulated when compared to aposymbiotic caeca cells. By contributing to host cuticle reinforcement, amino acid provision from the endosymbiont to the insect is crucial in the *Sitophilus-Sodalis* nutritional endosymbiosis [79]. According to previous metabolic pathway reconstructions based on the genome sequences, *S. pierantonius* supplies the host with four essential amino acids, namely arginine, lysine, phenylalanine and threonine [65, 66]. Here, we strengthen with transcriptomic data that the endosymbiont presence enhances the host metabolism of these amino acids in bacteriocytes when compared to aposymbiotic caeca cells. When comparing peripheral and central bacteriocytes with aposymbiotic caeca cells, we found that genes involved in the metabolism of various amino acids were upregulated, namely arginine (11 and 5 genes, respectively), lysine (3 and 3 genes), phenylalanine (2 and 1 genes) and threonine (4 and 1 genes,Fig. 3).

Besides genes related to the metabolism of these essential amino acids, we also found that genes involved in the tyrosine metabolism are upregulated in peripheral (8 genes) and central bacteriocytes (1 gene), when compared to aposymbiotic caeca cells, respectively (Fig. 3). Tyrosine can be synthesised by the host from the phenylalanine provided by the bacteria. It is a precursor of dihydroxy-phenylalanine (DOPA), which is crucial for cuticle sclerotization [79]. Tyrosine was not the only non-essential amino acid which metabolism seems to be upregulated in symbiotic bacteriocytes, when compared to aposymbiotic caeca. Less expected, we also found 13 aspartate and glutamate metabolism-related genes upregulated in peripheral bacteriocytes when compared to aposymbiotic caeca cells (Fig. 3). Among them, three were also upregulated in central bacteriocytes (Fig. 3). The aspartate and glutamate produced by the host can be used as precursors for three amino acids that the bacteria provide to the host: threonine, lysine and arginine, respectively [65, 66]. Hence, the upregulation of aspartate and glutamate metabolism in the host reveals a coordinated complementation of amino acid synthesis between weevils and endosymbionts inside the bacteriocytes, reinforcing the concept of “metabolic factories”, where the host and the endosymbiont complement their respective metabolic capacities [3, 37]. Likewise, Nakabachi and Ishikawa reported an increased expression of three host genes related to glutamate synthesis in the bacteriocytes of the young pea aphid *Acyrthosiphon pisum* [59]. The glutamate could be used as a precursor by the endosymbiont *Buchnera* to synthesise essential amino acids [59].

Apart from amino acid metabolism, we found a significant enrichment of GO terms related to fatty acid metabolism in bacteriocytes, when compared to aposymbiotic caeca cells (Fig. 2 and Table S6). This finding is in line with previous results indicating that aposymbiotic and symbiotic granary weevils (*Sitophilus granarius* L.) had different fatty acid profiles: while aposymbiotic weevils contained more palmitic, stearic, linoleic and linolenic acids, which are present in wheat grains, symbiotic weevils had higher levels of oleic, lauric and palmitoleic acid, suggesting that the endosymbiont impacts host fatty acid metabolism [83]. Fatty acids can serve as an energy source and are involved in insect reproduction and flight [76]. Interestingly, in addition to the well-described contribution to insect cuticle synthesis, the endosymbiotic status has also been shown to favour insect reproduction and flight ability, which are reduced or lost in aposymbiotic insects [31, 35]. We found that fatty acid metabolism-related genes, including an acyl-CoA synthetase, two aldehyde dehydrogenase, four elongation of very long chain fatty acid protein, a fatty acid synthase, a stearoyl-CoA desaturase, a very long-chain fatty acid—CoA ligase and 11 fatty acyl-CoA reductase (FAR) encoding genes, are upregulated in peripheral bacteriocytes, when compared to aposymbiotic caeca cells. FARs are needed for the reduction of fatty alcohols, which are precursors of sex pheromones [56], wax esters [38] and cuticular hydrocarbons [44]. Hydrocarbons constitute approximately 32% of the surface extracts of *S. oryzae* [6] and serve as waterproofing and signalling compounds in many insects [8]. RNAi against FAR encoding genes in the brown planthopper *Nilaparvata lugens* showed that certain FARs are essential for cuticle-shedding, hydrocarbon production and/or female fertility [45]. In addition to FAR encoding genes, we also found propanoate (4-aminobutyrate aminotransferase, acetyl-CoA synthetase, acyl-CoA synthetase, ethylmalonyl-CoA decarboxylase, methylmalonate-semialdehyde dehydrogenase and succinate–CoA ligase) and butanoate (2-hydroxyglutarate dehydrogenase, 3-hydroxybutyrate dehydrogenase and 4-aminobutyrate aminotransferase) metabolism-related genes upregulated in bacteriocytes as compared to aposymbiotic caeca cells. Propionate, together with methylmalonyl-CoA, are precursors of methyl-branched hydrocarbons [7]. Recently, it has been demonstrated that symbiotic weevils were more resistant to desiccation than aposymbiotic ones, although the authors did not demonstrate a change in the composition and quantity of cuticular hydrocarbons with the methods used in this work [40]. Consistent with these previous findings, our transcriptomic analysis suggests that endosymbionts may contribute to host cuticle reinforcement not only through amino acid provision, but also by influencing fatty acid production.

Overall, these data indicate that while carbohydrate metabolism (mainly carbohydrate degradation) is upregulated in aposymbiotic caeca cells, bacteriocytes in symbiotic insects exhibit enhanced fatty acid and amino acid metabolism, coordinated with the endosymbiont’s contributions to amino acid synthesis and influence on fatty acid metabolism. These findings highlight the role of the host transcriptome in transforming bacteriocytes into interkingdom “metabolic factories” [3, 37].

### The transcriptome of bacteriocytes reflects their polyploidy

To characterise the transcriptional signature of bacteriocytes compared to epithelial cells, we identified “common” differentially expressed genes (DEGs) in peripheral and central bacteriocytes compared to epithelial cells (Figure S2B). When compared to epithelial cells, 1139 and 1854 weevil genes were upregulated and downregulated in bacteriocytes, respectively (Figure S2B and Table S5).

**Fig. 4:**
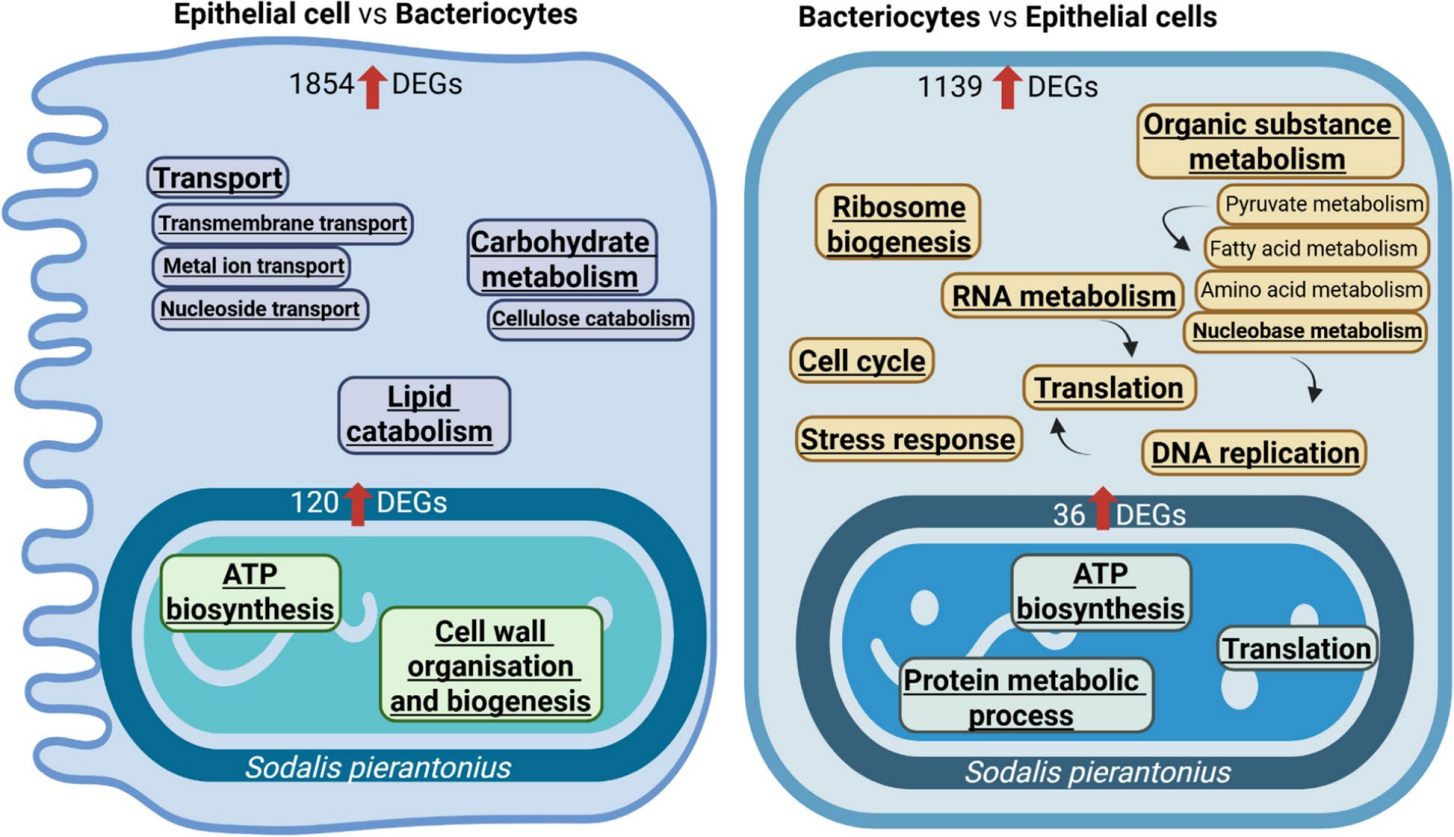
Distinct cell behaviour of both host and endosymbiont in bacteriocytes and epithelial cells. Enriched GO terms in upregulated genes in epithelial cells compared to central and peripheral bacteriocytes (left) and in central and peripheral bacteriocytes compared to epithelial cells (right). Underlined GO terms indicate significant functional enrichment (FDR adjusted-*P* ≤ 0.05)

**Fig. 5:**
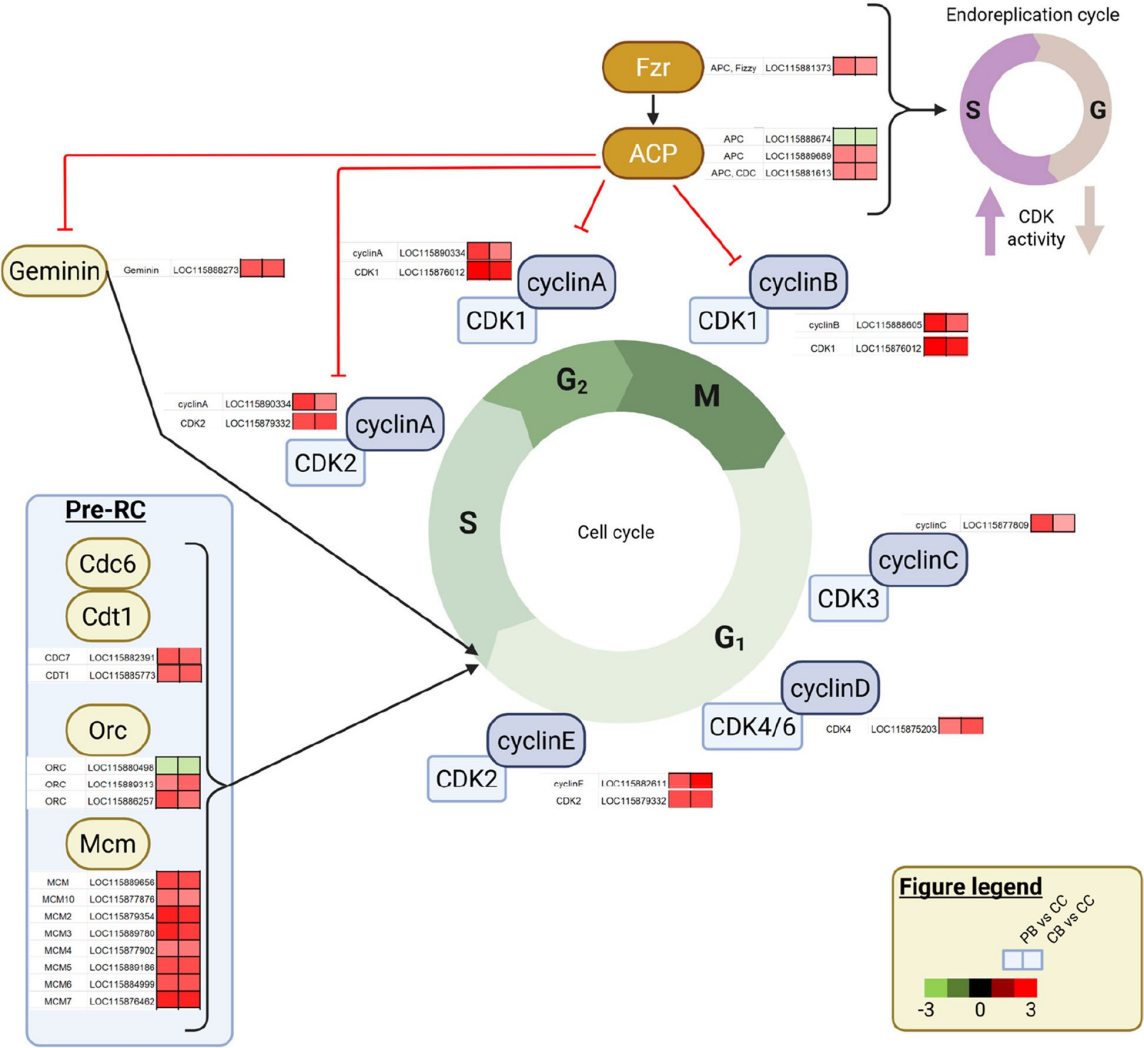
Regulation of the cell cycle and the endocycle switch, and the expression of orthologous genes in *Sitophilus oryzae* bacteriocytes. Differentially expressed genes from *Sitophilus oryzae* related to cell cycle and endocycle switch were identified in the pairwise comparisons between peripheral (PB) and central bacteriocytes (CB) compared to epithelial cells (EC). For each gene, two squares represent Log_2_-transformed fold change values of peripheral (left) and central bacteriocytes (right) compared to epithelial cells, according to the colour scale reported. Black and red lines indicate activation and inhibition, respectively. Figure created by compiling and adapting figures from Ventura and Giordano [77] and Ren et al. [67]. APC, anaphase promoting complex; CDC6, cell division cycle protein 6; CDK, cyclin-dependent kinase; CDT1, cell division cycle protein 10-dependent transcript 1 protein; Fzr, fizzy-related; MCM, minichromosome maintenance protein; ORC, origin recognition complex; Pre-RC, pre-replication complex

A significant GO enrichment for upregulated weevil genes in bacteriocytes compared to epithelial cells was found for cell cycle, DNA replication and organic substance metabolism, including nucleobase metabolism, ribosome biogenesis, RNA metabolism, stress response and translation (Fig. 4 and Table S6). More specifically, among genes involved in cell cycle regulations, we found eight cyclin- and six cyclin-dependent kinase (CDK) encoding genes upregulated in bacteriocytes, when compared to epithelial cells (Fig. 5 and Table S5). Cyclins form complexes with cyclin-dependent kinases (CDKs) that regulate cell cycle switches between G1, S, G2 and M phases [58]. Transcriptional repression of cyclins results in an endocycle onset, in which the cell cycle alternates only between S and G phases [29]. In the follicle cells and midgut cells of *Drosophila*, Fizzy-related (Fzr) regulates the anaphase promoting complex (APC), which initiates endocycle entry [16, 19]. Interestingly, we found three APC and one CDK inhibitor-encoding genes upregulated in bacteriocytes, when compared to epithelial cells, suggesting that bacteriocytes enter an endoreplication cycle. Endocycles generate cell polyploidy, as the cell undergoes multiple rounds of genome duplication in the absence of cell division, resulting in a single, enlarged, polyploid nucleus [85].

The maintenance of the endocycle at the G phase requires APC-mediated low CDK activity and the inhibition of Geminin, which ensures that one round of replication occurs during each cell cycle [24]. The low CDK activity allows the pre-replication complex (pre-RC) formation to initiate DNA replication in the S phase. In the S phase, the high CDK activity promotes DNA synthesis while inhibiting the pre-RC formation [29]. Here, we found several pre-replication complex protein-encoding genes upregulated in bacteriocytes compared to epithelial cells, which indicates that DNA replication could be maintained in bacteriocytes (Fig. 5).

Taken together, these results suggest a polyploid nature of bacteriocytes as Nardon suggested [62]. Polyploidy enables tissue growth and massive production of molecules at a low biological cost by circumventing the time, metabolites, and energy required to complete mitotic cycles [23, 51]. Polyploidy has been previously reported in endosymbiont harbouring cells and organs [49, 53, 61, 64]. Bacteriocyte polyploidy increases the cell size, accommodating a higher number of bacteria, and increases the production of metabolites, including precursors used by the endosymbionts, hence resulting in a globally increased metabolic capacity. Moreover, an interesting feature of polyploid cells is that DNA replication does not necessarily involve doubling the whole genome; the over replication of specific regions could generate a robust gene expression related to specialised cell function [29].

### Endosymbionts have distinct transcriptional behaviours in the different host cell types

To address whether the endosymbionts express diverse transcriptional programs according to the cell types in which they reside, we identified endosymbiont DEGs in the pairwise comparisons between bacteriocytes and epithelial cells. While we did not find any DEGs when comparing *S. pierantonius* expression in peripheral and central bacteriocytes, we found that 36 and 120 *S. pierantonius* genes were upregulated and downregulated in bacteriocytes compared to epithelial cells, respectively (Figure S2C and Table S7). Upregulated *S. pierantonius* genes in bacteriocytes compared to epithelial cells showed a significant GO enrichment for ATP biosynthesis, protein metabolic process and translation (Fig. 4 and Table S8). As for upregulated *S. pierantonius* genes in epithelial cells compared to bacteriocytes, a significant GO enrichment was found for ATP biosynthesis and cell wall organisation and biogenesis (Fig. 4 and Table S8). We found an UDP-N-acetylmuramate dehydrogenases encoding gene (MurB; SOPEG_3602), UDP-N-acetylglucosaminyl 1-phosphate transferase (SOPEG_0383) involved in cell wall biosynthesis and a β-hexosaminidase (SOPEG_1778) and lipoprotein NlpD (SOPEG_1030) involved in peptidoglycan recycling, upregulated in the bacteria present in epithelial cells, when compared to the bacteria located in the peripheral bacteriocytes. This suggests that endosymbionts undergo more extensive cell wall remodelling, likely associated with cell division, in epithelial cells, when compared to bacteriocytes. Interestingly, these cell wall organisation and biogenesis genes were not significantly differentially expressed between epithelial cells and central bacteriocytes nor between central and peripheral bacteriocytes, but only between peripheral bacteriocytes and epithelial cells. This suggests that the endosymbionts residing in central bacteriocytes show an intermediate transcriptional program compared to the endosymbionts residing in epithelial cells and those in peripheral bacteriocytes, at least in terms of genes involved in cell wall remodelling. A weevil antimicrobial peptide (AMP), ColA has been shown to prevent endosymbiont escape from bacteriocytes and inhibit bacterial cell division without affecting DNA replication, resulting in the formation of gigantic, polyploid, metabolically active bacterial cells [46]. Consistent with these findings, this transcriptomic analysis reveals significantly higher expression of *colA* (LOC115874620) in peripheral bacteriocytes, when compared to epithelial cells. This suggests that ColA inhibits the cell wall remodelling necessary for cytokinesis in bacteriocytes, while in epithelial cells, where *colA* expression is lower, endosymbionts can replicate with reduced interference. In addition to *colA*, we identified upregulation of four other AMP genes (*defensin*, *diptericin-1*, *diptericin-2* and *diptericin-like*) in peripheral bacteriocytes, when compared to epithelial cells, suggesting their potential role in controlling endosymbionts. Similarly, the Diptericin-like AMP encoding gene was shown to be correlated with bacterial load during development [28].

These results indicate that endosymbionts do not have the same transcriptional behaviour in the different cell types, likely responding to host factors such as ColA. While endosymbionts present in bacteriocytes show a transcriptomic signature evocative of a high metabolic state (ATP biosynthesis, protein metabolic process and translation), the endosymbionts in epithelial cells show instead a profile evocative of cell divisions, inhibited by the host factor ColA in bacteriocytes. Altogether the parallel analysis of the host and endosymbiont transcriptomes in different cell types illustrate a coordinated switch toward metabolic complementation of the two partners in the bacteriocytes.

### The transcriptome of central bacteriocytes indicates their role in vesicle-mediated transport

**Fig. 6:**
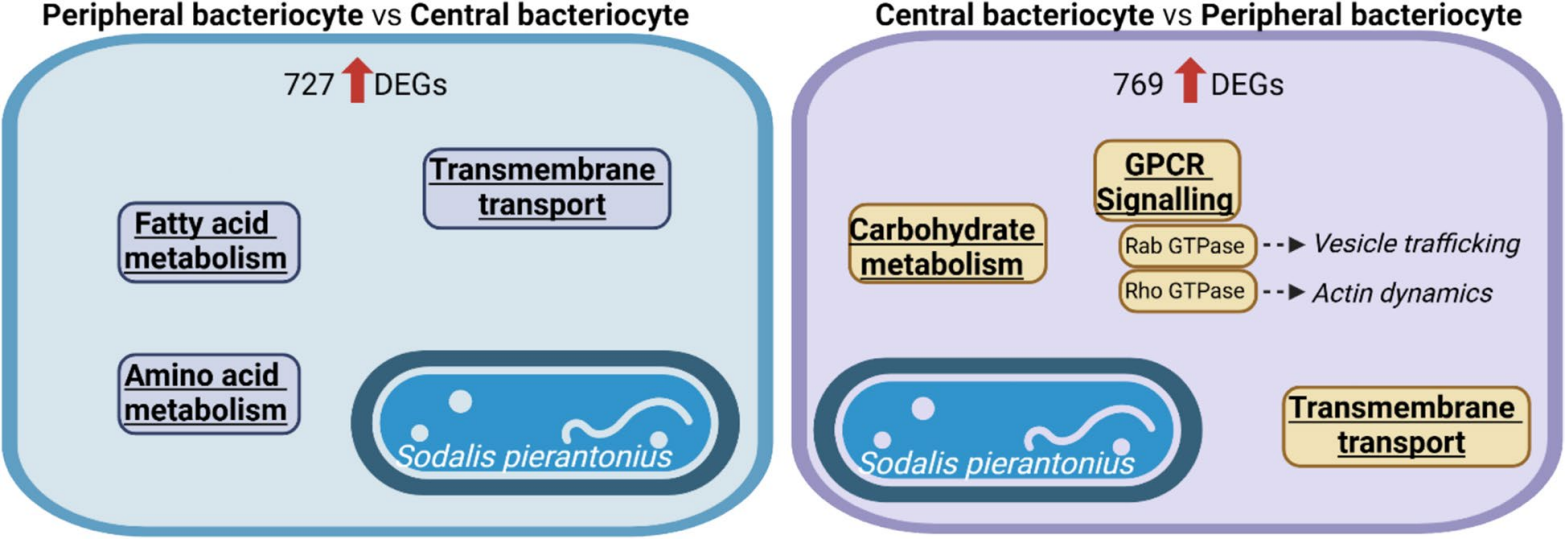
Cellular specialisation of peripheral and central bacteriocytes. Enriched GO terms in upregulated host genes in peripheral bacteriocytes compared to central bacteriocytes (left) and in central bacteriocytes compared to peripheral bacteriocytes (right). Underlined GO terms indicate significant functional enrichment (FDR adjusted-*P* ≤ 0.05)

To address the functional specialisation of peripheral and central bacteriocytes, we compared their gene expression profiles. In the pairwise comparison, 727 and 769 weevil genes are upregulated and downregulated in peripheral bacteriocytes, compared to central bacteriocytes, respectively (Figure S2D and Table S5). Upregulated genes in peripheral bacteriocytes compared to central bacteriocytes showed significant GO enrichment for transmembrane transport, fatty acid metabolism and amino acid metabolism. This indicates that between central and peripheral bacteriocytes, the latter are more metabolically active in the pathways in relation to the endosymbiont metabolic contribution to the host (amino acid and fatty acid metabolism; Fig. 6 and Table S6). This is in line with the fact that peripheral bacteriocytes are harbouring the majority of endosymbionts (Fig. 1C–D), hence more precursors are likely available for the host to use in amino acid and fatty acid metabolism in these cells.

As for central bacteriocytes compared to peripheral bacteriocytes, upregulated genes showed GO enrichment for categories related to transmembrane transport, carbohydrate metabolism and G-protein coupled receptor signalling (GPCR signalling; Fig. 6 and Table S6).

G-proteins are highly conserved molecular switches regulating many fundamental cellular processes by transducing the signal from activated GPCRs at the cell membrane to various intracellular signalling cascades [10]. G-proteins are GTPases that switch between the active guanosine 5′-triphosphate (GTP)-bound after their activation by a GPCR and the inactive guanosine diphosphate (GDP)-bound states. We found that 11 Rho GTPase-encoding genes (RhoA, RhoL, Rho GTPase, seven RhoGEF and Rhophilin) are upregulated in central bacteriocytes as compared to peripheral bacteriocytes. Rho GTPases are involved in actin cytoskeleton organisation, cell adhesion and phagocytosis [32]. Transcription factors, such as Myc, have been shown to induce RhoA encoding gene transcription [13]. We found that five Myc transcription factors are upregulated in central bacteriocytes, when compared to peripheral bacteriocytes. Besides Rho GTPase-encoding genes, we found that six Rab GTPase (four unspecified Rab GTPase, Rab3 and Rab32) and two ARF GTPase (ARF guanyl-nucleotide exchange factor and arfaptin) encoding genes were upregulated in central bacteriocytes, when compared to peripheral bacteriocytes. Moreover, we also found microtubule-encoding genes (e.g. Dynein, Formin, Kazrin, Nesprin, Tau and Titin) upregulated in central compared to peripheral bacteriocytes. Both Rab and ARF GTPases are associated with microtubule-dependent vesicle trafficking [22, 72]. Cells can internalise a variety of materials, including metabolites, carbohydrates, proteins, lipids, DNA or RNA through endocytosis, then endocytic vesicles undergo maturation from early to late endosomes during which process they are sorted and disseminated to their destination [25, 39].

**Fig. 7:**
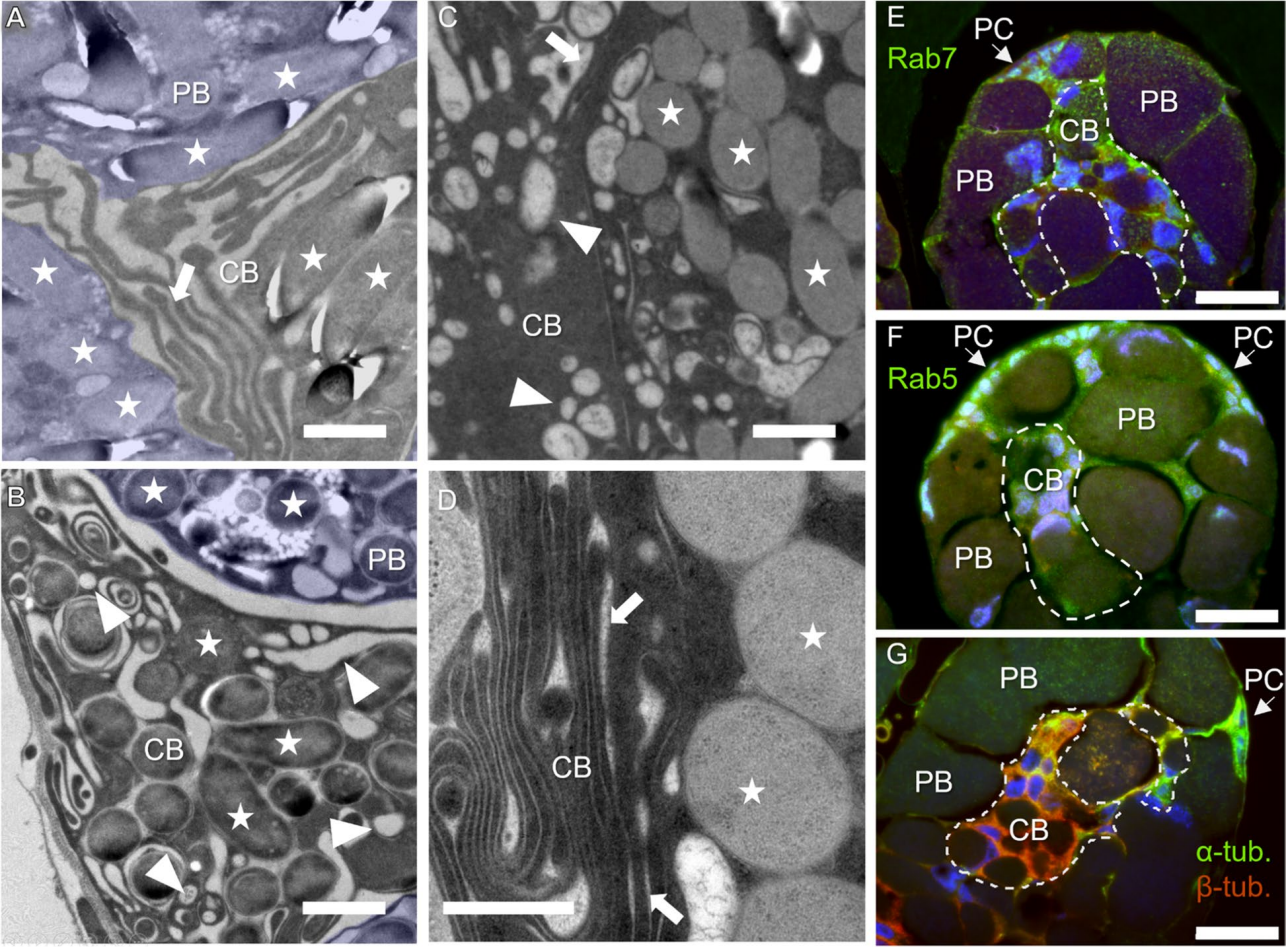
Membranous structures in central bacteriocytes. **A–D** Ultrastructure of central bacteriocytes (CB) observed with TEM. Peripheral bacteriocytes (PB) are covered with a blue layer. Examples of bacteria are indicated with stars. Membranous structures and vesicular-like structures are indicated with arrows and arrowheads, respectively. **E–G** Immunostaining of the bacteriome with antibodies targeting markers for late endosome Rab7 (**E**), early endosomes Rab5 (**F**), and microtubule-components α-tubulin and β-tubulin (**G**), respectively. **E–F** Transversal section. **G** Sagittal section with a view of the basis of the caecum. Arrows indicate progenitor cells (PC) Blue: DAPI. Green: Rab7 (**E**), Rab5 (**F**), α-tubulin (**G**). Red: autofluorescence (**E**, **F**), β-tubulin (**G**). The magenta colour in (**F**) results from the combination of the red (autofluorescence) and blue (DAPI) signals. The yellow colour in (**G**) results from the combination of the red (β-tubulin) and green (α-tubulin) signals. Scale bars: 1 µm (**A–C**), 0.5 µm (**D**), and 30 µm (**E–G**)

To further investigate the function of central bacteriocytes, we explored their ultrastructural organisation, by performing transmission electron microscopy (TEM) upon high pressure cryofixation (HPF) of the tissues, a procedure that preserves the lipidic membranes. The imaging revealed that the central bacteriocytes contain numerous intracellular vesicles, as well as complex membranous protrusions extending in between neighbouring peripheral bacteriocytes (Fig. 7A–D). To better characterise the vesicles, present in central bacteriocytes, we used immunostaining performed on paraffin sections with antibodies raised against early (Rab5) and late (Rab7) endosome markers and against microtubule-components, the α- and β-tubulin. The results strengthen that central bacteriocytes display high amounts of early and late endosomes. Endosomes seem less numerous in peripheral bacteriocytes, although late endosomes can be seen in the distal border of these cells (corresponding to the more external region of the bacteriome organ), suggesting a possible function in nutrient and/or information exchange with the rest of the organism (Fig. 7E–F). Central bacteriocytes display a significant labelling of α- and β-tubulin (Fig. 7G), reinforcing the idea of an active, microtubule-dependent vesicular transport in these cells. Besides central bacteriocytes, we also found that progenitor cells display α-tubulin labelling, likely related to their active division (Fig. 7G).

Intracellular vesicle trafficking may ensure different functions related to the endosymbiotic interaction, including nutrient transport and signalling from host cells to host cells, and/or from host cells to endosymbionts. Moreover, although data are lacking to ascertain that extracellular vesicles are produced by the bacteriocytes in this system, in addition to the intracellular vesicles observed, it is an interesting possibility, as such vesicles have been proposed to mediate microbe-host communication in a wide variety of associations [26].

Upregulation of Rab GTPases and ARF GTPases encoding genes in central bacteriocytes suggests the specialisation of these cells in exchanging material and/or information through vesicle trafficking with surrounding cells. This assumption is in accordance with previous results, suggesting that Rab GTPase could be involved in membrane transport in the *Sitophilus-Sodalis* endosymbiosis [36]. More specifically, a Rab GTPase encoding gene was found to be upregulated in the weevil larval bacteriome, when compared to aposymbiotic larvae, and its role in cellular traffic was speculated [36]. Rab GTPase also regulates phagocytosis and autophagy [73], which could play a role in establishing, regulating and recycling endosymbionts [78]. Likewise, in pea aphid-*Buchnera* endosymbiosis, other Rab GTPase encoding genes were found to be highly expressed in bacteriocytes. The authors speculated their involvement in mediating intracellular trafficking [60], and the degradation of the endosymbiont *Buchnera* in ageing aphids [70]. Endocytic/exocytic activity related genes were also found upregulated in adult versus nymphal bacteriocytes in the whitefly (*Bemisia tabaci*) and the authors speculated their involvement in vesicle-mediated translocation of nutrients or signals [48].

Taken together, the upregulation of microtubule and Rab-encoding genes on one hand, and Rho encoding genes on the other hand, stresses the importance of both the microtubule and actin cytoskeleton for the function of central bacteriocytes. Further research is needed to understand the content, trajectory and function of these vesicles in this endosymbiotic association.

### Nutritional stress induces complex transcriptional reprogramming in peripheral bacteriocytes and associated endosymbionts

The description of the precise transcriptional program of the different cell types and their endosymbionts raises the question of to which extent these transcriptional programs are dependent on the diet and/or host-endosymbiont specific. To address the plasticity of nutritional endosymbiosis, we applied nutritional stress and studied the transcriptional response of both host and endosymbiont. Treatments were chosen to mimic drastic changes in dietary conditions. Starch diet was used to mimic a pure, and therefore highly unbalanced, carbohydrate diet, in comparison to the regular grain diet of the cereal weevils, which contains approximately 70% of starch, but also proteins and traces of lipids and minerals [71]. Starvation mimicked nutrient scarcity. Briefly, newly emerged adults were allowed to feed on a grain diet (GR), on pure carbohydrate, starch diet (ST) or kept under starvation (N) for 2 days before we performed dual RNA-seq on microdissected peripheral bacteriocytes from 3-day-old adult insects. We choose to focus on peripheral bacteriocytes *i.e.* the cells where the host and endosymbiont metabolism is highly integrated, as confirmed by the transcriptomic analysis above.

Sequencing results are summarised in Table S2. Briefly, from 68.39 to 122.39 million weevil reads and 4.82 and 38.66 million of endosymbiont reads were obtained for each replicate of peripheral bacteriocytes collected from insects under different feeding protocols (Table S2). Among the total reads obtained in peripheral bacteriocytes from insects kept on grain diet, from 22.18 to 23.55% were endosymbiont reads (Figure S3A and Table S2). Endosymbiont reads were higher in bacteriocytes from insects kept on starch (17.51 to 29.16% of total reads), confirming previous results that weevils kept on starch diet present higher endosymbiont load, when compared to weevils fed on grain [18]. In contrast, endosymbiont reads account for only 3.56 to 4.60% of the total reads found in peripheral bacteriocytes from insects kept under starvation, in line with previous results that endosymbiont load is lower under starved conditions [18]. This difference in the bacterial load is also visible when using FISH. While the morphology of mesenteric caeca was similar between weevils kept on grain and starch diet (Figure S4A–B), fewer and smaller peripheral bacteriocytes were observed in weevils kept under starvation (Figure S4C). Weevil (Figure S3B) and endosymbiont (Figure S3C) expression profiles revealed clear distinction under nutritional stress. This indicates that both host and endosymbiont react to nutritional stress.

### Carbohydrate intake is sufficient to maintain amino acid but not fatty acid metabolism

**Fig. 8:**
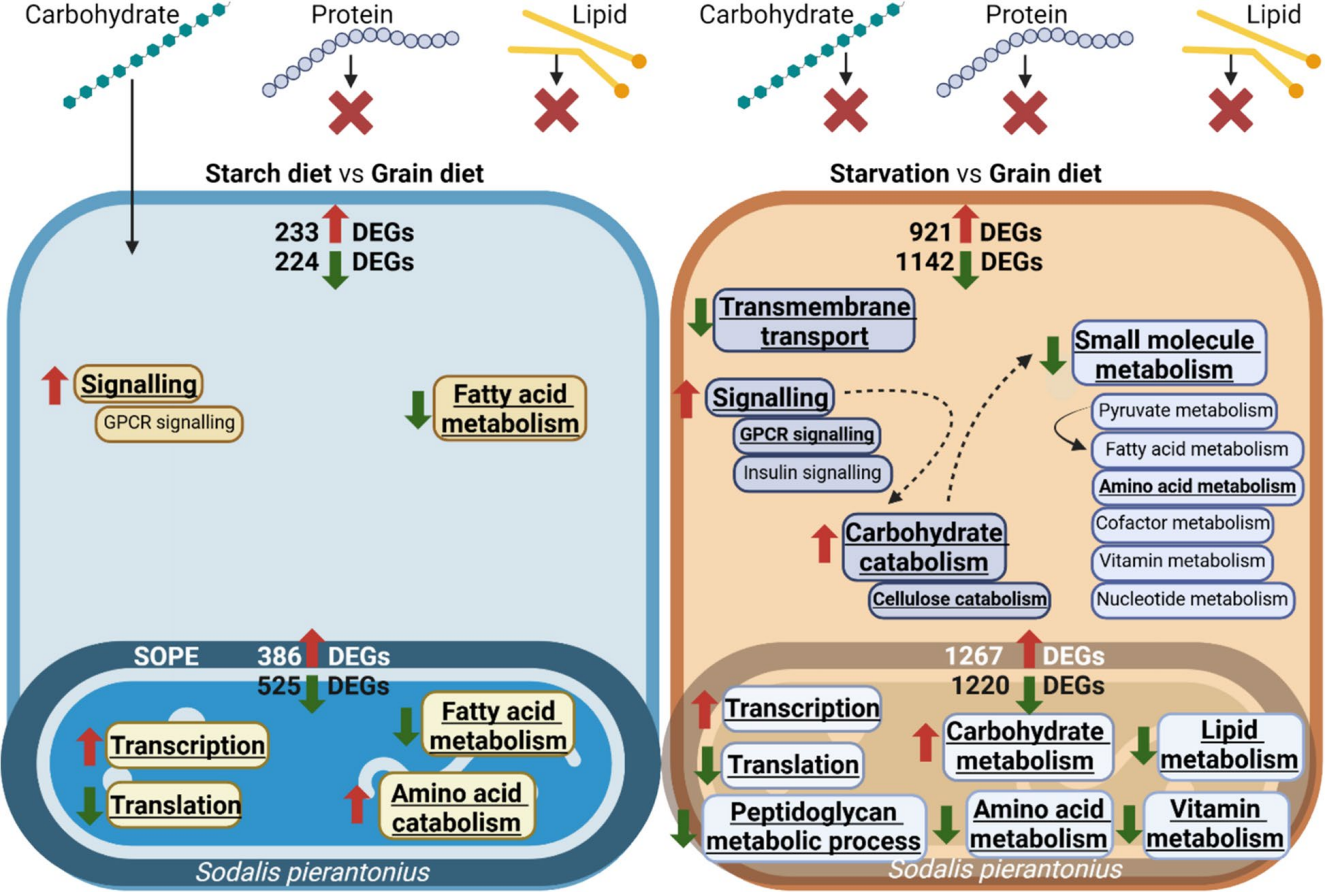
Host and endosymbiont strongly respond to nutritional challenges. Enriched GO terms in upregulated (red arrows) and downregulated (green arrows) genes in *Sitophilus oryzae* peripheral bacteriocytes and in *Sodalis pierantonius* residing in them, kept on grain versus starch diet (left) or grain diet versus starvation (right). Underlined GO terms indicate significant functional enrichment (FDR adjusted-*P* ≤ 0.05)

We first compared the transcriptomes of the bacteriocytes and endosymbionts of weevils fed on standard grain diet or on starch-only diet. Both host and endosymbiont strongly responded to changes in diet composition. In peripheral bacteriocytes of weevils kept on starch compared to grain diet, 233 and 224 weevil genes were upregulated and downregulated, respectively (Figure S5A and Table S5). A significant enrichment of GO terms related to signalling and fatty acid metabolism was found for upregulated and downregulated DEGs, respectively, in peripheral bacteriocytes of weevils kept on starch compared to grain diet (Fig. 8 and Table S6).

**Fig. 9:**
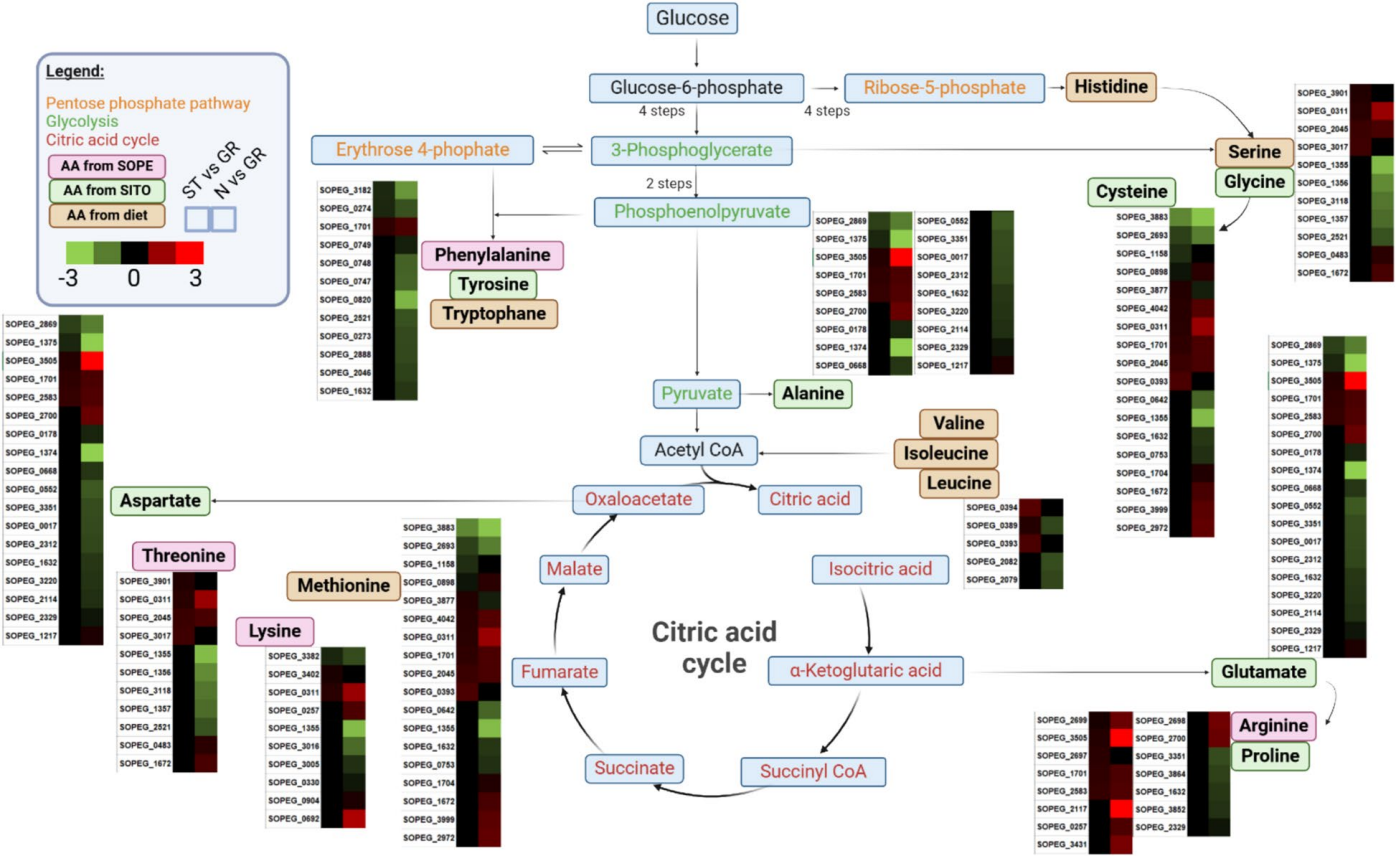
Endosymbiont amino acid metabolism is affected by changes in diet. Differentially expressed genes related to amino acid (AA) metabolism from *Sodalis pierantonius*, residing in peripheral bacteriocytes. For each gene, two squares represent Log_2_-transformed fold change values of the pairwise comparisons between peripheral bacteriocytes from insects kept on grain versus starch (left) or grain versus starvation (right), according to the colour scale reported. Pink, green and brown boxes indicate amino acids provided by *Sodalis pierantonius* (SOPE)*, Sitophilus oryzae* (SITO) and obtained from diet, respectively

As for the endosymbiont, we found 386 and 525 genes upregulated and downregulated, respectively, in peripheral bacteriocytes of weevils kept on starch diet compared to grain diet (Figure S5B and Table S7). We found significant enrichment in translation and fatty acid metabolism for the downregulated DEGs and enrichment in transcription and amino acid catabolism for upregulated DEGs (Fig. 8 and Table S8). Starch diet positively affected the metabolism of most amino acids as evaluated by the number of related genes (Fig. 9). This increased amino acid metabolism from the endosymbiont side could contribute to host adaptation to extremely unbalanced dietetic conditions. These results are in accordance with previous findings that endosymbiont presence is essential under unbalanced diet, which was demonstrated by total mortality of aposymbiotic weevils kept on starch diet, as compared to symbiotic weevils [18]. Only the genes related to the metabolically costly aromatic amino acid metabolism were negatively affected under starch diet, suggesting that under unbalanced diet endosymbionts might provide less phenylalanine. This could explain the results of Dell’Aglio et al. [18], where endosymbionts are maintained longer in young adults under this type of diet. Taken together, a carbohydrate-only diet reduces the efficacy of aromatic amino acid provision from the endosymbiont side, resulting in a longer cuticle synthesis, and consequently a longer maintenance of endosymbionts.

In contrast to amino acid metabolism, we found that both host and endosymbiont fatty acid metabolism are negatively affected under starch diet. Hence, while we have shown evidence that under standard grain diet endosymbionts could contribute to host fatty acid metabolism (see above), this contribution may be lost when the diet is strongly unbalanced and lacks fatty acids. These results show the transcriptional plasticity of *Sitophilus-Sodalis* endosymbiosis depending on the diet. The ability of *Sodalis* to partially compensate for a heavily unbalanced diet could lie in the recent nature of the *Sitophilus-Sodalis* endosymbiotic association [43]. Indeed, the genome of *S. pierantonius* has retained many genes [65] and the ability to regulate its gene expression [28, 52], in contrast with ancient endosymbionts [33, 69, 74]. In line with this, low transcriptional plasticity was reported in ancient endosymbioses in response to changes in diet, as a result of the extreme gene loss in the endosymbiont and the decreased ability to regulate their own gene expression [54, 55, 68]. Hence the endosymbiont transcriptional plasticity observed here is likely in relation with the endosymbiont relatively recent acquisition by the host *Sitophilus* and could represent an advantage for the host in terms of adaptation to changing quality in the diet conditions.

We then analysed the impact of starvation on the bacteriocyte and endosymbiont transcriptomes. Starvation resulted in the upregulation and downregulation of 921 and 1142 weevil genes in peripheral bacteriocytes, respectively, when compared to grain diet (Figure S5A and Table S5). Host upregulated genes showed GO enrichment for signalling, including G-protein coupled signalling and carbohydrate catabolism (Fig. 8 and Table S6), while downregulated genes showed enrichment for transmembrane transport and small molecule metabolism, including amino acid metabolism (Fig. 8 and Table S6). These results are in agreement with the starvation response described in insects converting carbohydrate and lipid resources to energy [84]. For example, we found carbohydrate catabolism (*e.g.* α-amylase, α-glucosidase, α-L-fucosidase, β-mannosidase, β-galactosidase, endoglucanase, maltase, polygalacturonase, pectinesterase) related genes upregulated under starvation. Observation with transmission electron microscopy (TEM) indicated the presence of early (Figure S6A–C) and late-stage autophagy figures (Figure S6D–F); however, we were unable to demonstrate a more massive autophagy under starvation, as compared to grain and starch-diets.

Interestingly, the endosymbiont is reacting strongly to starvation by modulating the expression of an unusually high number of genes. We found 1267 and 1220 endosymbiont genes upregulated and downregulated, respectively, in response to starvation (Figure S5B and Table S7). Upregulated endosymbiont genes showed an enrichment for transcription and carbohydrate metabolism (Fig. 8 and Table S8). Downregulated endosymbiont genes showed enrichment for translation, peptidoglycan metabolic process, amino acid, lipid and vitamin metabolism (Fig. 8 and Table S8). Under starvation, *S. pierantonius* is unable to maintain its amino acid metabolism, as evaluated by the number of DEGs and expression level (Fig. 9). This contrasts with the endosymbiont response to starch-diet, characterised by an increase in amino acid metabolism. Interestingly, the endosymbiont morphology changed under starvation, as observed by electron microscopy. The majority of endosymbionts appeared swollen, and their membrane seemed damaged, as compared with endosymbionts in insects under grain and starch-diets, which appear rather similar (Figure S6G–I).

These data suggest a high cost in terms of carbohydrates from the endosymbiont part under the host starvation, coupled with a reduced input from the endosymbiont to the insect, which could explain the increase in mortality previously observed for symbiotic weevils starved for two days after their final ecdysis, while aposymbiotic weevils were unaffected [18]. Under starvation conditions, maintaining endosymbionts becomes a burden rather than an advantage, as they consume host resources, ultimately reducing insect fitness.

Taken together, our results show the transcriptional plasticity of *Sitophilus-Sodalis* endosymbiosis on changing diets. Endosymbionts seem to compensate for an unbalanced, starch-only diet, through an increase in the metabolism of certain amino acids. This illustrates that in more recent endosymbiosis, such as the *Sitophilus-Sodalis* association, there is still room for some “metabolic negotiations” between partners that can favour the host adaptation to changes in diet composition [9]. In more extreme conditions, under starvation, there is no room for such “metabolic negotiations” and the endosymbionts become a metabolic burden rather than an advantage, as they compete for the remaining energy source without contributing to amino acid synthesis.

## Conclusion

In summary, we show in this study how the morphologically different cell types constituting the mesenteric caecum express distinct transcriptional programs. We show that cell-specific response to endosymbionts in bacteriocytes compared to aposymbiotic midgut cells included the upregulation of fatty acid and amino acid metabolism, likely enhancing their metabolic potential and potentially manifesting in host fitness benefits, such as reproduction, developmental time and flight ability. As aposymbiotic insects possess two cell types (epithelial cells and caeca cell) in their mesenteric caecum with distinct transcriptional profile, our results do not confirm previous assumptions that bacteriocytes are epithelial cells whose cell destiny has been modified by the presence of endosymbionts. Moreover, the large size, gigantic nuclei, high metabolic potential together with enhanced DNA metabolism in bacteriocytes, as compared to epithelial cells, suggest their polyploid nature. We hypothesise that polyploidy could contribute to increased metabolic activity of endosymbiont harbouring cells by increasing both the cell size to house bacteria and the cell metabolism. We characterised central bacteriocytes as smaller cells, which seem to be in direct contact both with the gut lumen, for the most basal of them inside the bacteriome organ, and with the peripheral bacteriocytes that surround them. Central bacteriocytes display numerous intracellular vesicles that were stained with Rab5 and Rab7 antibodies, and numerous membranous extensions. We found Rho GTPase, Rab GTPases and tubulin-encoding genes upregulated in these cells as compared to peripheral bacteriocytes, indicating the importance of both the actin cytoskeleton and the microtubule cytoskeleton-based vesicle transport in these cells. These results suggest a signalling and/or transport function for these cells. One hypothesis could be a function in the transport of nutrients from the gut lumen to the peripheral bacteriocytes, where the metabolic activity of both the host and the endosymbiont is massive. Further analysis of the content of the vesicles and their trafficking pattern will be needed to test this hypothesis. Our results also raise the question of the ontogeny of these two types of bacteriocytes. The data presented here do not allow to discriminate whether central and peripheral bacteriocytes are two distinct cell types originating from the same progenitor cells, or whether they could represent two stages of differentiation of the same cell type. In this case, central bacteriocytes could represent an intermediate stage between de novo infected progenitor cells that are expanding to accommodate the increasing number of endosymbionts, and the fully infected and metabolically more active peripheral bacteriocytes. Further studies will be needed to address these hypotheses. Finally, our data demonstrate that diet composition strongly impacts both host and endosymbiont transcriptomes and show a molecular trade-off among metabolic pathways. While under starch diet endosymbionts succeed in maintaining amino acid metabolism, endosymbionts become a burden to the host under starvation. Taken together, this study shows how the metabolic potential of weevil midgut mesenteric caeca cells is enhanced by the presence of endosymbionts and highlights key genes involved in their metabolic performance.

## Supplementary Information

Additional file 1: Figure S1. Host and endosymbiont transcriptomic signatures in different cell types. Figure S2. Host and endosymbiont differentially expressed genes in the different cell types. Figure S3. Host and endosymbiont transcriptomic signatures in peripheral bacteriocytes of weevils under different diets. Figure S4. Mesenteric caecum morphology under nutritional stress. Figure S5. Host and endosymbiont differentially expressed genes in peripheral bacteriocytes from insects kept on different diets. Figure S6. Bacteriocyte and endosymbiont morphology in insects kept under different diets.

Additional file 2: Table S1. Antibodies used for immunostaining. Table S2. Dual RNA sequencing and mapping results for each replicate. Table S3. Expression levels of *Sitophilus oryzae* genes in the different cell types. Table S4. Expression levels of *Sodalis pierantonius* genes in the different cell types. Table S5. Differentially expressed genes (DEGs) of *Sitophilus oryzae* identified in the different cell types. Table S6. Enrichment analysis on differentially expressed genes (DEGs) of *Sitophilus oryzae* identified in the different cell types. Table S7. Differentially expressed genes (DEGs) of *Sodalis pierantonius* identified in the different cell types. Table S8. Enrichment analysis on differentially expressed genes (DEGs) of *Sodalis pierantonius* identified in the different cell types.

## Data Availability

Raw sequencing data from this study have been deposited at the National Center of Biotechnology Information (NCBI) Sequence Read Archive (SRA) under PRJNA1195835.
