# Supplementary material for: Dual-transcriptomics on microdissected cells reveals functional specialisation of symbiont-bearing-cells and contrasted responses to nutritional stress in the cereal weevil
Source: Microbiome. 2025 Aug 6;13:182. doi: 10.1186/s40168-025-02164-0 (PMC12326831; doi:10.1186/s40168-025-02164-0)
Supplement: Supplementary file 2 — Additional file 1: Figure S1. Host and endosymbiont transcriptomic signatures in different cell types. Figure S2. Host and endosymbiont differentially expressed genes in the different cell types. Figure S3. Host and endosymbiont transcriptomic signatures in peripheral bacteriocytes of weevils under different diets. Figure S4. Mesenteric caecum morphology under nutritional stress. Figure S5. Host and endosymbiont differentially expressed genes in peripheral bacteriocytes from insects kept on different diets. Figure S6. Bacteriocyte and endosymbiont morphology in insects kept under different diets. [file 40168_2025_2164_MOESM1_ESM.docx]

**
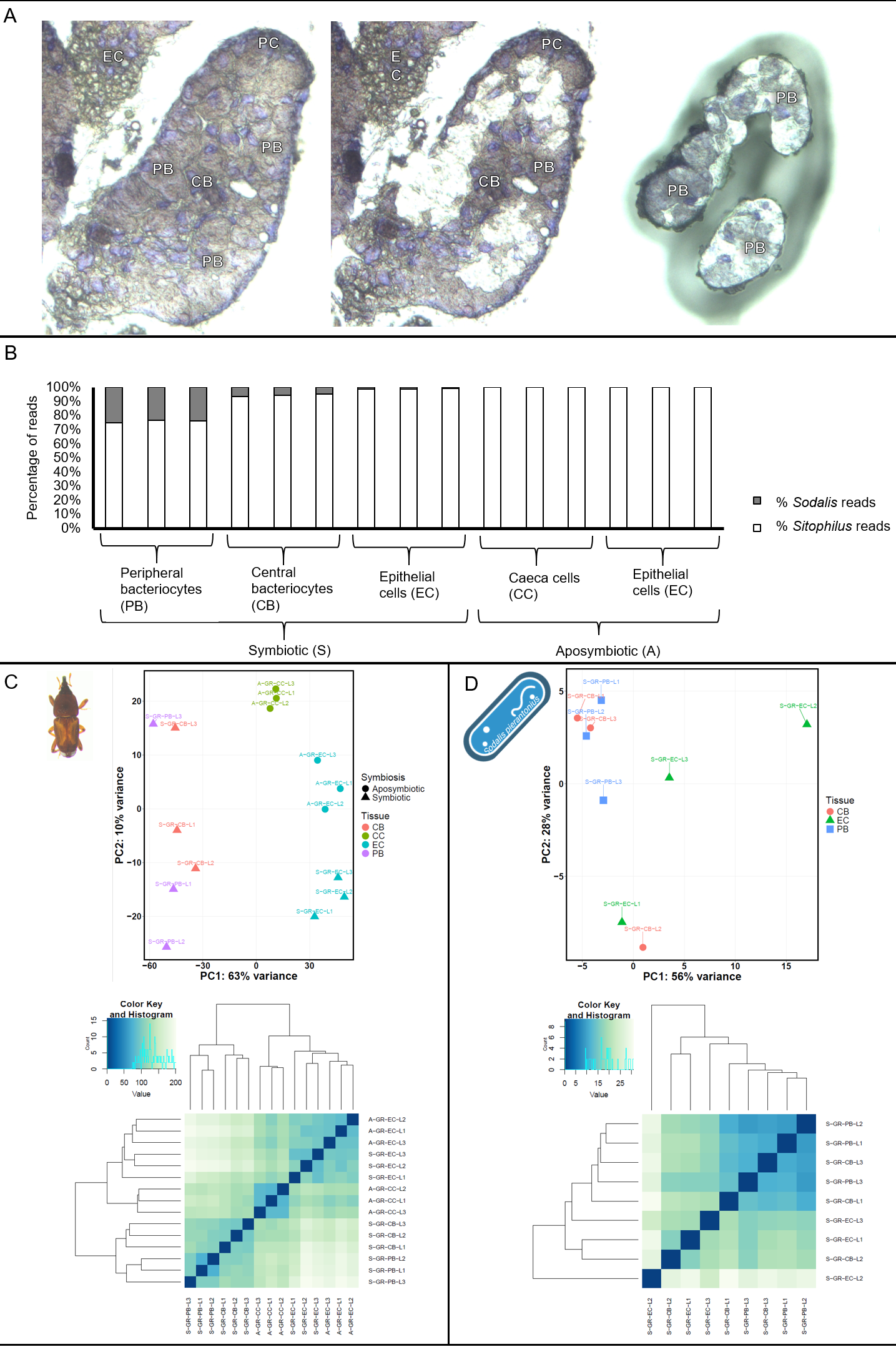
Figure S1: Host and endosymbiont transcriptomic signatures in different cell types. (A)** Example of LCM capture. **(B)** Percentage of reads mapping to *Sitophilus oryzae* and *Sodalis pierantonius* genome. **(C-D)** PCA (up) and hierarchical (down) clustering of *Sitophilus oryzae* (C) and *Sodalis pierantonius* (D) reads obtained from different cell types. From symbiotic insects (S), *Sitophilus oryzae* and *Sodalis pierantonius* reads were obtained from peripheral bacteriocytes (PB) central bacteriocytes (CB) epithelial cells (EC). From aposymbiotic insects (A), *Sitophilus oryzae* reads were obtained from caeca cells (CC) and epithelial cells (EC).

**
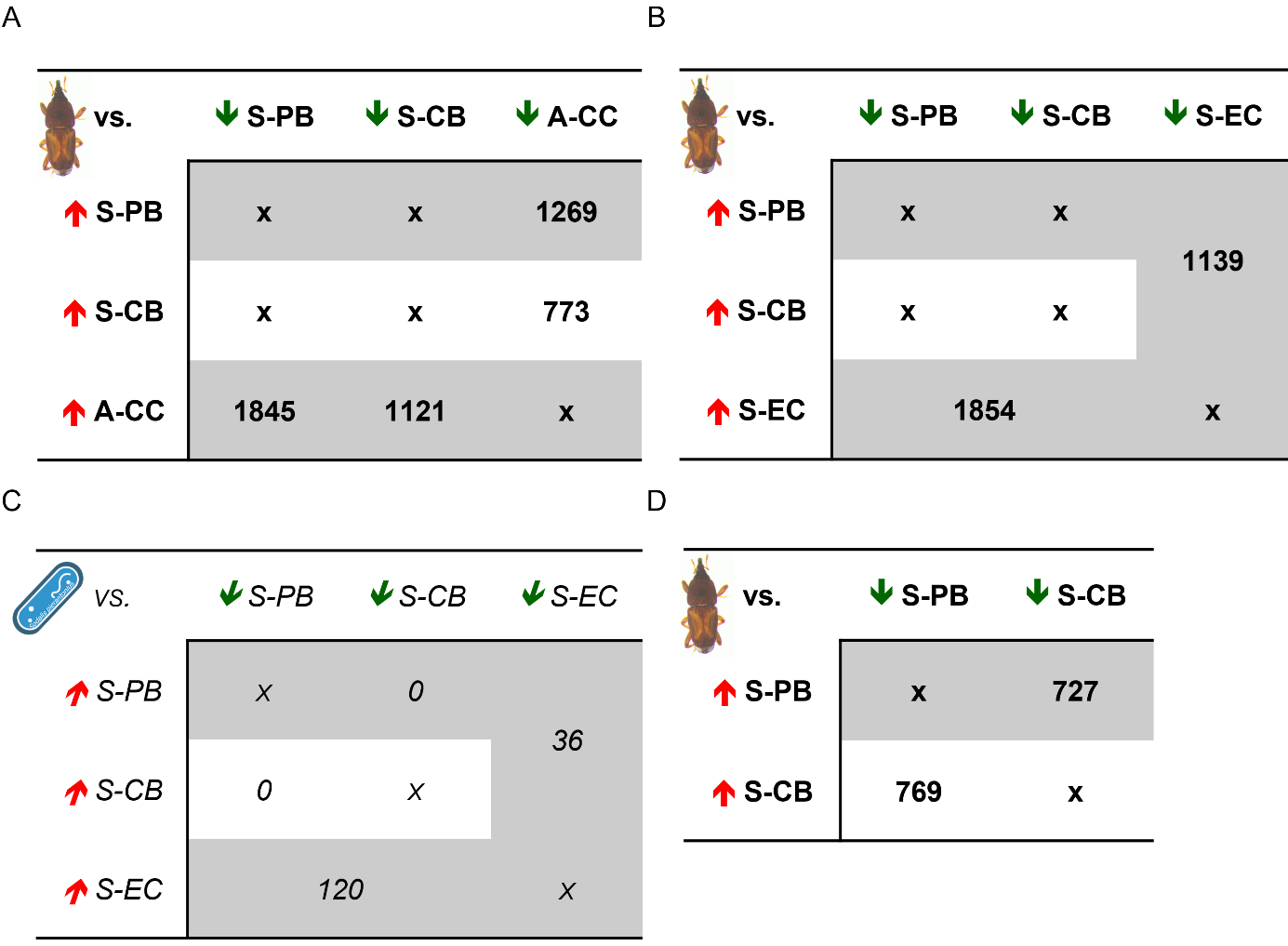
Figure S2: Host and endosymbiont differentially expressed genes in the different cell types. (A)** *Sitophilus oryzae* differentially expressed genes in peripheral bacteriocytes (S-PB) and central bacteriocytes (S-CB) versus aposymbiotic caeca cells (A-CC). **(B)** Differentially expressed genes of *Sitophilus oryzae* and **(C)** *Sodalis pierantonius* (in italics) in bacteriocytes (S-PB and S-CB) versus epithelial cells (S-EC). **(D)** Differentially expressed genes of *Sitophilus oryzae* in peripheral bacteriocytes (S-PB) versus central bacteriocytes (S-CB). Red arrows: upregulated genes; green arrows: downregulated genes.

**
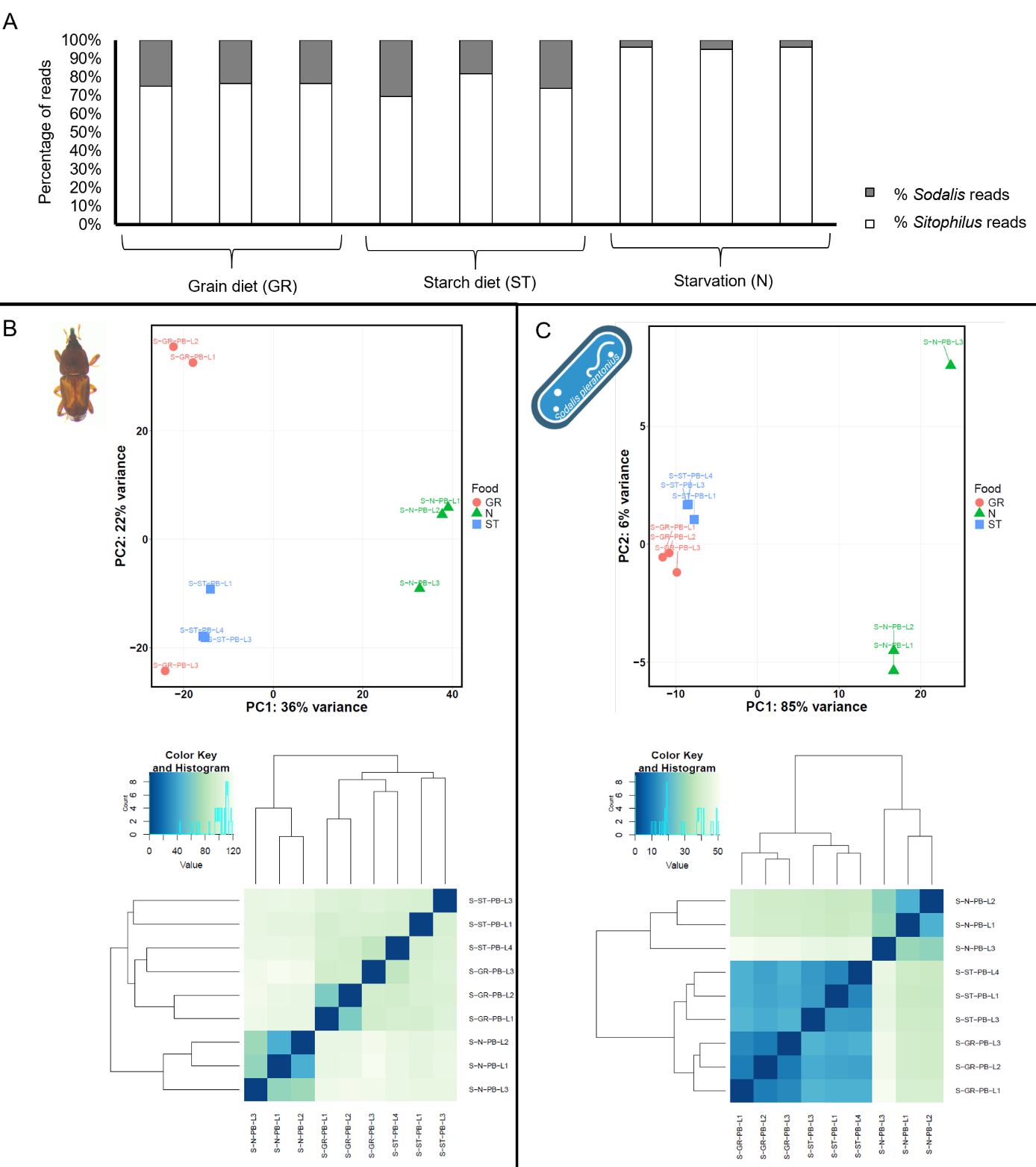
Figure S3: Host and endosymbiont transcriptomic signatures in peripheral bacteriocytes of weevils under different diets. (A)** Percentage of reads mapping to *Sitophilus oryzae* and *Sodalis pierantonius* genome. **(B-C)** PCA (up) and hierarchical (down) clustering of *Sitophilus oryzae* (B) and *Sodalis pierantonius* (C) reads obtained from different cell types. *Sitophilus oryzae* and *Sodalis pierantonius* reads were obtained from peripheral bacteriocytes (PB) of insects kept under grain diet (GR), starch diet (ST) or under starvation (N).


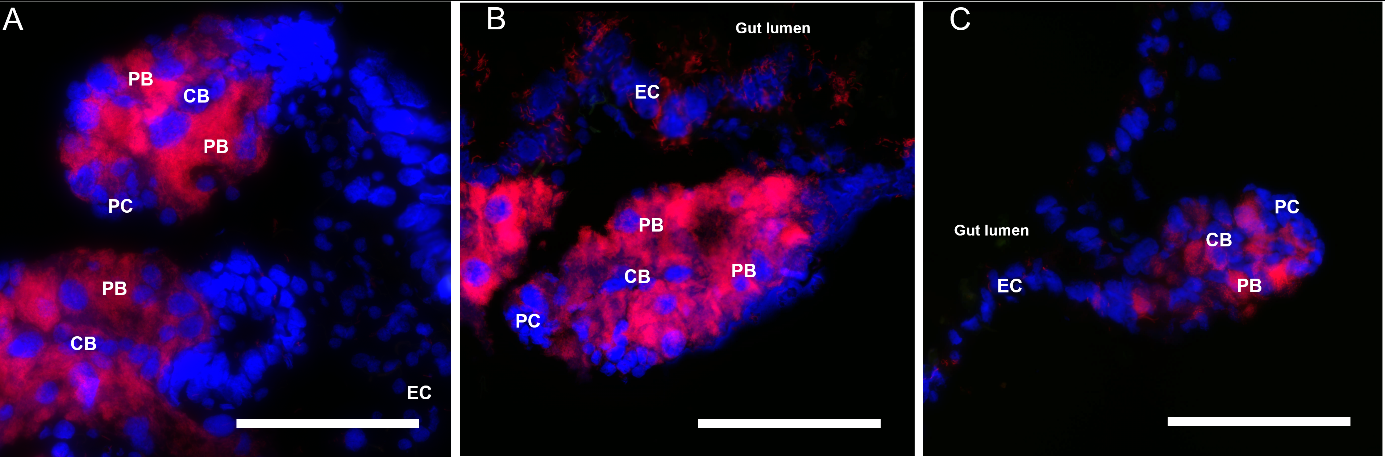


**Figure S4. Mesenteric caecum morphology under nutritional stress (A-C)** Mesenteric caeca and bacteriomes from symbiotic insects kept on grain diet (A) starch diet (B) and under starvation (C) stained with FISH probe targeting the 16S rRNA of *Sodalis pierantonius*. Progenitor cells (PC), peripheric bacteriocytes (PB), central bacteriocytes (CB), and epithelial cells (EC) forming the basis of the mesenteric caecum. Scale bars: 100 µm.

**
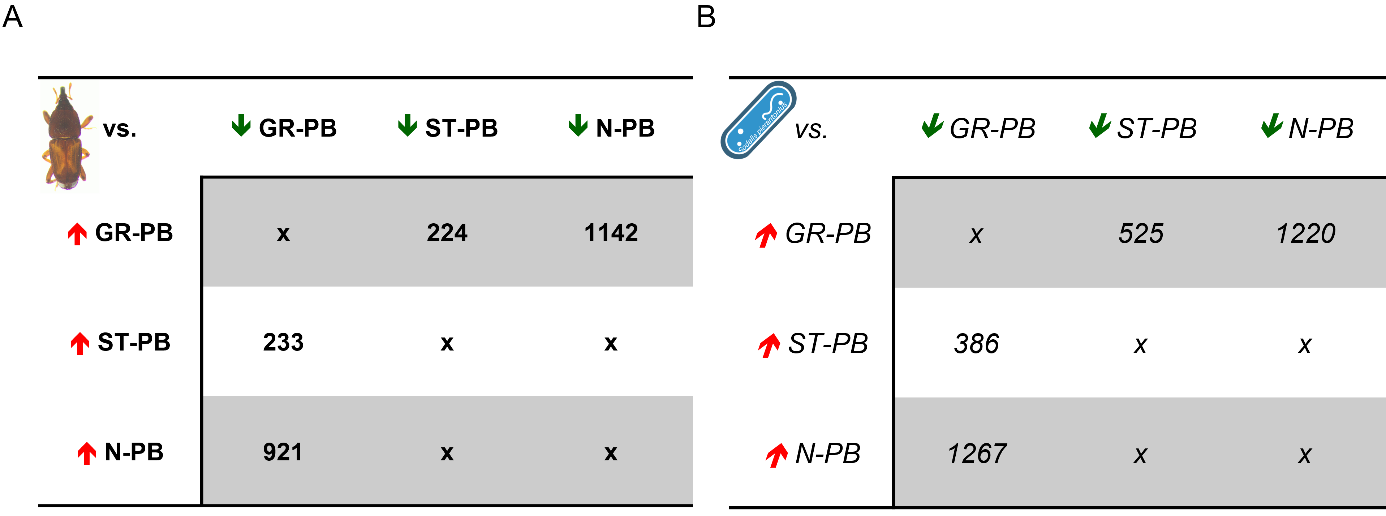
Figure S5: Host and endosymbiont differentially expressed genes in peripheral bacteriocytes from insects kept on different diets. (A)** *Sitophilus oryzae* differentially expressed genes in peripheral bacteriocytes (PB) from insects kept on grain (GR) versus starch diet (ST) or on grain diet versus starvation (N). **(B)** *Sodalis pierantonius* (in italics) differentially expressed genes in peripheral bacteriocytes from insects kept on grain versus starch diet or on grain diet versus starvation. Red arrows: upregulated genes; green arrows: downregulated genes.

**
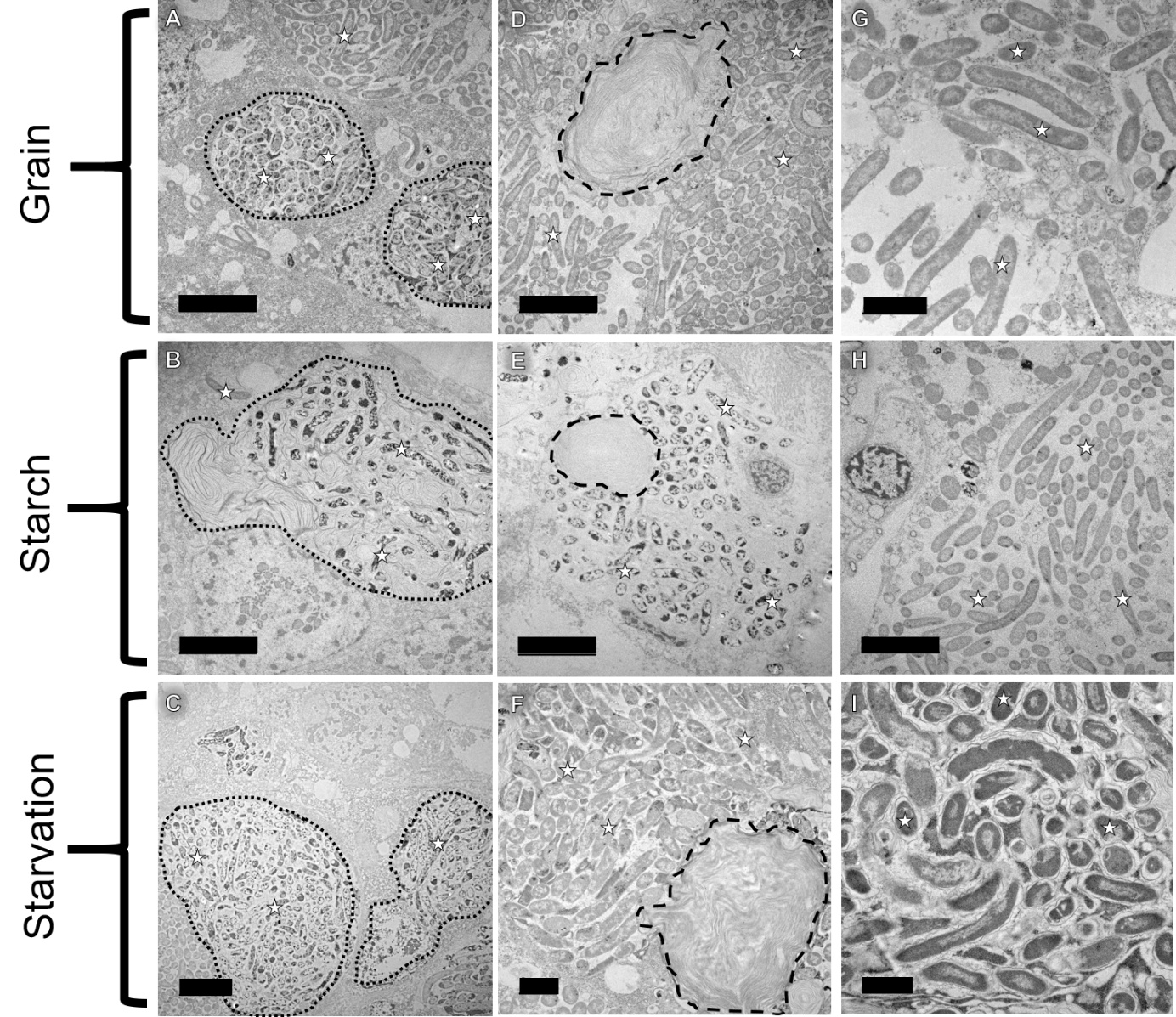
Figure S6. Bacteriocyte and endosymbiont morphology in insects kept under different diets.** Transmission electron microscopy observations on peripheral bacteriocytes from insects kept on grain, starch diet and under starvation. **(A-C)** Early autophagy figures indicated with dotted lines. **(D-F)** Late autophagy figures indicated with dashed lines. **(G-I)** Endosymbiont morphology. Examples of bacteria are indicated with asterisks. Scale bars: 5 µm (A-E and H) 2 µm (F and G) and 1 µm (I).

**Legend of Supplementary Excel Tables:**

**Table S1. Antibodies used for immunostaining.** Primary antibody (column A). Antibody description (column B). Antibody host (column C). Dilution used (column D). Secondary antibody (column E).

**Table S2. Dual RNA sequencing and mapping results for each replicate.** Peripheral bacteriocytes (PB), central bacteriocytes (CB) and epithelial cells (EC) from symbiotic insects (S) kept on grain diet (GR) starch diet (ST) and under starvation (N) and caeca cells (CC) and epithelial cells (EC) from aposymbiotic insects (A) kept on grain diet (GR) were analysed in triplicate by Dual RNA-Seq (column A-F). Read pairs were obtained by Dual RNA-Seq sequencing (column G) and read pairs that passed the quality check are reported in column H. Read pairs mapped to unique locations to the *Sitophilus oryzae* genome and the corresponding percentage (%) of filtered read pairs are reported (column I-J). Read pairs mapped to unique locations to the *Sodalis pierantonius* genome and the corresponding percentage (%) of filtered read pairs are reported (column K-L). Treatment code indicates symbiotic status (S- for symbiotic; A-for aposymbiotic) diet (GR: grain diet; ST: starch diet; N: no diet, starvation) and cell types (PB: peripheral bacteriocytes; CB: central bacteriocytes; EC: epithelial cells; CC: caeca cells).

**Table S3. Expression levels of *Sitophilus oryzae* genes in the different cell types.** Gene expression levels, assessed as Log10(TPM) values in peripheral bacteriocytes (PB), central bacteriocytes (CB) and epithelial cells (EP) from symbiotic insects (S) kept on grain diet (GR), starch diet (ST) and under starvation (N) and in caeca cells (CC) and epithelial cells (EC) from aposymbiotic insects (A) kept on grain diet (GR). *Sitophilus oryzae* gene identifier (column A) and protein description (column B) from the Soryzae_2.0 release (https://www.ncbi.nlm.nih.gov/datasets/genome/GCF_002938485.1/). Mean expression levels (column C-I). Expresssion level of each replicate (column J-AD). Treatment code indicates symbiotic status (S- for symbiotic; A-for aposymbiotic) diet (GR: grain diet; ST: starch diet; N: no diet, starvation) and cell types (PB: peripheral bacteriocytes; CB: central bacteriocytes; EC: epithelial cells; CC: caeca cells).

**Table S4. Expression levels of *Sodalis pierantonius* genes in the different cell types.** Gene expression levels, assessed as Log10(TPM) values in peripheral bacteriocytes (PB), central bacteriocytes (CB) and epithelial cells (EC) from symbiotic insects (S) kept on grain diet (GR), starch diet (ST) and under starvation (N). *Sodalis pierantonius* gene identifier (column A) and protein description (column B) from the ASM51740v1 release (https://www.ncbi.nlm.nih.gov/datasets/genome/GCF_000517405.1/). Mean expression levels (column C-G). Expresssion level of each replicate (column H-V). Treatment code indicates symbiotic status (S- for symbiotic; A-for aposymbiotic) diet (GR: grain diet; ST: starch diet; N: no diet, starvation) and cell types (PB: peripheral bacteriocytes; CB: central bacteriocytes; EC: epithelial cells; CC: caeca cells).

**Table S5. Differentially expressed genes (DEGs) of *Sitophilus oryzae* identified in the different cell types.** Fold change values of *Sitophilus oryzae* DEGs identified in pairwise comparisons between peripheral bacteriocytes (PB), central bacteriocytes (CB) and epithelial cells (EC) from symbiotic insects (S) kept on grain diet (GR), starch diet (ST) and under starvation (N) and in caeca cells (CC) and epithelial cells (EC) from aposymbiotic insects (A) kept on grain diet (GR). *Sitophilus oryzae* gene identifier (Soryzae_2.0; column A), Log2-transformed fold change values (column B-J) and P values (column K-S) calculated for each pairwise comparisons between different cell types. DEGs are in bold (P ≤ 0.05 and Log2 (fold change) ≥1 or ≤ -1). Genes discussed in the manuscript are marked in bold. Protein description (column T), Clusters of Otrhologous genes (column U and Y-Z), Gene ontology terms (column V) and Kyoto Encyclopedia of Genes and Genomes Pathway terms (column W) of the Soryzae_2.0 release (https://www.ncbi.nlm.nih.gov/datasets/genome/GCF_002938485.1). Treatment code indicates symbiotic status (S- for symbiotic; A-for aposymbiotic) diet (GR: grain diet; ST: starch diet; N: no diet, starvation) and cell types (PB: peripheral bacteriocytes; CB: central bacteriocytes; EC: epithelial cells; CC: caeca cells).

**Table S6. Enrichment analysis on differentially expressed genes (DEGs) of *Sitophilus oryzae* identified in the different cell types.** Enrichment analysis on *Sitophilus oryzae* DEGs identified in pairwise comparisons between peripheral bacteriocytes (PB), central bacteriocytes (CB) and epithelial cells (EC) from symbiotic insects (S) kept on grain diet (GR), starch diet (ST) or under starvation (N) and in caeca cells (CC) and epithelial cells (EC) from aposymbiotic insects (A) kept on grain diet (GR). Gene ontology terms for biological processes (column A-F) for molecular function (column G-L) and for cellular components (column M-R) identified with G-profiler (https://biit.cs.ut.ee/gprofiler/gost). Gene ontology terms (column A-B; G-H and M-N), number of genes annotated to a GO term in the *Sitophilus oryzae* genome Soryzae_2.0 release (https://www.ncbi.nlm.nih.gov/datasets/genome/GCF_002938485.1; columns C; I and O), number of DEGs belonging to each GO term (column D; J and P) and P values (column E; K and Q) identified in a pairwise comparison (column F; L and R). Treatment code indicates symbiotic status (S- for symbiotic; A-for aposymbiotic) diet (GR: grain diet; ST: starch diet; N: no diet, starvation) and cell types (PB: peripheral bacteriocytes; CB: central bacteriocytes; EC: epithelial cells; CC: caeca cells).

**Table S7. Differentially expressed genes (DEGs) of *Sodalis pierantonius* identified in the different cell types.** Fold change values of *Sodalis pierantonius* DEGs identified in pairwise comparisons between peripheral bacteriocytes (PB), central bacteriocytes (CB) and epithelial cells (EC) from symbiotic insects (S) kept on grain diet (GR), starch diet (ST) or under starvation (N). *Sodalis pierantonius* gene identifier (ASM51740v1; column A), Log2-transformed fold change values (column B-F) and P values (column G-K) calculated for each pairwise comparisons between different cell types. DEGs are in bold (P ≤ 0.05 and Log2 (fold change) ≥0.2 or ≤ -0.2). Genes discussed in the manuscript are marked in bold. Protein description (column L), Clusters of Orhologous genes (column M), Gene ontology terms (column N) and Kyoto Encyclopedia of Genes and Genomes Pathway terms (column O) of the ASM51740v1 release (https://www.ncbi.nlm.nih.gov/datasets/genome/GCF_000517405.1/). Treatment code indicates symbiotic status (S- for symbiotic; A-for aposymbiotic) diet (GR: grain diet; ST: starch diet; N: no diet, starvation) and cell types (PB: peripheral bacteriocytes; CB: central bacteriocytes; EC: epithelial cells; CC: caeca cells).

**Table S8. Enrichment analysis on differentially expressed genes (DEGs) of *Sodalis pierantonius* identified in the different cell types.** Enrichment analysis on *Sodalis pierantonius* DEGs identified in pairwise comparisons between peripheral bacteriocytes (PB), central bacteriocytes (CB) and epithelial cells (EC) from symbiotic insects (S) kept on grain diet (GR), starch diet (ST) and under starvation (N). Gene ontology terms for biological processes (column A-F) for molecular function (columns G-L) and for cellular components (column M-R) identified with topGO classic algorithm. Gene ontology terms (column A-B; G-H and M-N), number of genes annotated to a GO term in the *Sodalis pierantonius* genome from the ASM51740v1 release (https://www.ncbi.nlm.nih.gov/datasets/genome/GCF_000517405.1; columns C; I and O), number of DEGs belonging to each GO term (column D; J and P), P values (column E; K and Q) and identified in a pairwise comparison (column F; L and R). Treatment code indicates symbiotic status (S- for symbiotic; A-for aposymbiotic) diet (GR: grain diet; ST: starch diet; N: no diet, starvation) and cell types (PB: peripheral bacteriocytes; CB: central bacteriocytes; EC: epithelial cells; CC: caeca cells).
